# Genome‐wide association analyses reveal the genetic basis of combining ability in rice

**DOI:** 10.1111/pbi.13134

**Published:** 2019-04-29

**Authors:** Junxiao Chen, Hao Zhou, Weibo Xie, Duo Xia, Guanjun Gao, Qinglu Zhang, Gongwei Wang, Xingming Lian, Jinghua Xiao, Yuqing He

**Affiliations:** ^1^ National Key Laboratory of Crop Genetic Improvement and National Centre of Plant Gene Research (Wuhan) Huazhong Agricultural University Wuhan China

**Keywords:** combining ability, GCA, SCA, GWAS, heterosis, *Oryza sativa* L

## Abstract

Combining ability is a measure for selecting elite parents and predicting hybrid performance in plant breeding. However, the genetic basis of combining ability remains unclear and a global view of combining ability from diverse mating designs is lacking. We developed a North Carolina II (NCII) population of 96 *Oryza sativa* and four male sterile lines to identify parents of greatest value for hybrid rice production. Statistical analyses indicated that general combining ability (GCA) and specific combining ability (SCA) contributed variously to different agronomic traits. In a genome‐wide association study (GWAS) of agronomic traits, GCA and SCA, we identified 34 significant associations (*P *<* *2.39 × 10^−7^). The superior alleles of GCA loci (*Ghd8*,* GS3* and *qSSR4*) accumulated in parental lines with high GCA and explained 30.03% of GCA variance in grain yield, indicating that molecular breeding of high GCA parental lines is feasible. The distinct distributions of these QTLs contributed to the differentiation of parental GCA in subpopulations. GWAS of SCA identified 12 more loci that showed dominance on corresponding agronomic traits. We conclude that the accumulation of superior GCA and SCA alleles is an important contributor to heterosis and QTLs that greatly contributed to combining ability in our study would accelerate the identification of elite inbred lines and breeding of super hybrids.

## Introduction

Heterosis, or hybrid vigour, is a common biological phenomenon that refers to the superior performance of a hybrid (usually F_1_) relative to its parents (Darwin, 1876; Shull, 1908, 1914). The key issue for successful use of heterosis is to identify parents having potential for producing hybrid combinations with higher performance. However, parents with superior agronomic traits do not always pass those traits on to their progenies. To address this problem, combining ability has been employed in selecting parents and specific combinations in hybrid plant breeding (Ahangar *et al*., 2008; Comstock *et al*., 1949; Singh *et al*., 1974; Sprague and Tatum, 1942; Townsend *et al*., 2013; Williams *et al*., 1995; Wu and Matheson, 2004).

The concept of general combining ability (GCA) and specific combining ability (SCA) was first defined by Sprague and Tatum (1942) and clarified by Griffing (1956). GCA indicates the average performance of a parental line in hybrid combinations and reflects additive gene action. SCA is used to designate those cases in which certain combinations do relatively better or worse than would be expected on the basis of GCA of the lines involved and reflects nonadditive gene action. By utilizing mating designs such as NCII or diallel crossing, breeders can with ease identify parental lines with high GCA and combinations with high SCA (Comstock and Robinson, 1948). As most studies on combining ability aim at identifying elite parental lines, the genetic basis of combining ability is largely unknown (Lv *et al*., 2012). It is important to uncover the genetic basis of combining ability and improve the breeding of elite hybrid varieties.

Recently, the quantitative trait locus (QTL) mapping approach was adopted to study the genetic basis of GCA (Liu *et al*., 2015; Qi, 2013; Qu, 2012). Qu *et al*. developed a QTL mapping method for dissecting GCA of agronomic traits using three testcross populations and a backcross recombination inbred line (BCRIL) in rice. Their results indicated that GCA is also a kind of quantitative trait controlled by poly‐genes just like most mapped QTLs. Liu *et al*. (2015) utilized an F_2_‐based NCII design with five testers and identified 13 QTLs that accounted for the GCA of three yield‐related traits in rice. Their study firstly linked GCA of heading date to two known genes, *OsPRR37* and *Ghd7*. However, the QTL mapping approach could not dissect the genetic basis of SCA and the mapping population also lacked genetic diversity. A global view of the genetic bases of both GCA and SCA from a diverse parental mating design is lacking.

Early types of molecular markers were unable to meet the demands of researchers on combining ability in mating designs involving large numbers of parents. Second‐generation sequencing technologies enable re‐sequencing of large numbers of genomes and provide the possibility of high‐throughput genotyping and large‐scale surveys of genetic variation (Weigel and Mott, 2009). Sequence‐based genome‐wide association studies (GWASs) greatly contribute in dissecting the genetic architecture of complex agronomic and metabolic traits (Chen *et al*., 2014; Yano *et al*., 2016). Identification of allelic variation underpinning combining ability in rice will have enormous practical implications in rice breeding. In this study, we constructed a NCII population of 96 diverse cultivated rice and four male sterile lines to explore the genetic basis of combining ability and clarify the relationship between combining ability and hybrid performance. We performed GWAS to the GCA and SCA of 12 agronomic traits to identify important genes for hybrid rice breeding and improvement.

## Results

### Population structure and LD of the NCII population

**Figure 1:**
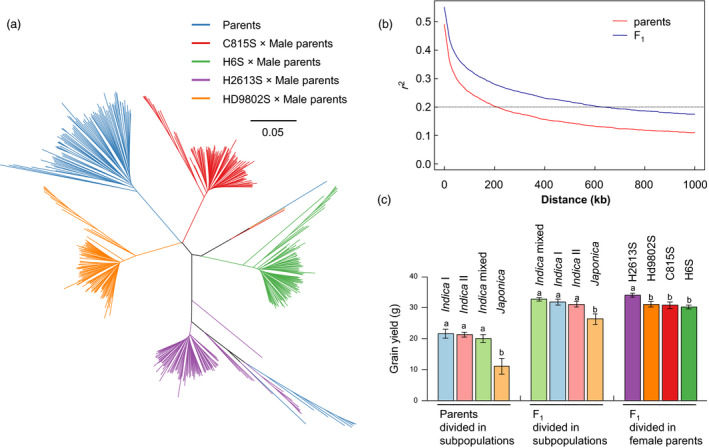
Genealogy, LD and grain yield of the population. (a) Neighbour‐joining tree of 100 parental lines and 384 F_1_ in our NCII population. (b) LD decay in parents and F_1_. (c) Comparison of grain yield of parents and F_1_ in different subpopulations.

The population used for studying combining ability contained 96 male parental lines, four female parental lines and four groups of F_1_ plants (Figure 1a and Figure S1a). Population structure analysis of the parental lines divided the male parents into eight *japonica* (Jap), 19 *indica I* (IndI), 46 *indica II* (IndII) and 23 *indica* intermediate (Ind) at *K* = 3 (Figure S1a, b). The four female parents were identified as *indica* intermediate. Principal component (PC) analysis revealed that *indica–japonica* differentiation explained most of the genetic variance, followed by differentiation among *indica* group (Figure S1c).

The population structure in the F_1_ generation showed some differences (Figure S2). The *indica–japonica* differentiation also contributed most to the genetic variance, but the genetic differences among female parents contributed more than the differentiation among *indica* group. Population structure analysis divided the F_1_ population into one group of *indica–japonica* hybrids and four groups of *indica–indica* hybrids at *K* = 5 (Figure S2a). In PC analysis, the first PC identified the *indica–japonica* hybrids, and the following PCs distinguished *indica–indica* hybrids from different female parents (Figure S2b).

As a strict inbreeding species, rice has a slow LD decay rate that reduces the resolution of GWAS. In our population, average LD did not decay below an *r* ^*2*^ value of 0.2, or ~200 kb in parents and 600 kb in F_1_ hybrids (Figure 1b), meaning that the non‐random mating greatly increased LD level and changed the population structure.

### Effects of population structure on agronomic traits

The average grain yield of F_1_ hybrids (mean 31.20 g per plant) was much higher than that of the parents (mean 20.18 g per plant), indicating high heterosis levels for grain yield. We calculated the phenotypic correlations among agronomic traits and found that relationships of the traits in hybrids were consistent with those of the parents (Figure S3a). Yield is a complicated trait determined by a combination of panicle number, flowers per panicle, seed setting rate and grain weight, but there are often negative correlations among the traits.

Yield traits also showed distinct distributions among subpopulations. Historically, many of the highest yielding varieties were bred within *indica*; not surprisingly, *indica* outperformed *japonica* in several components of yield such as panicle number, seed setting rate, grain number and grain yield (Fujita *et al*., 2013; Hedden, 2003). However, subspecies differences significantly decreased from parents to hybrids presumably due to heterotic effects (Figure 1c and Figures S3b and S4a). *Indica*–*japonica* hybrids had the highest levels of heterosis, but their yield performances were still lower than those of *indica*–*indica* hybrids. This may be caused by the hybrid sterility between *indica* and *japonica* (Yang *et al*., 2012a).

Maternal genetic effects largely contribute to population structure and to phenotypic variation in the F_1_ generation. The progenies of H2613S had the highest average grain yield (34.06 g) across the four female parents, indicating that H2613S had the highest GCA (Figure 1c and Figure S4b). H2613S is a derivative of Guangzhan 63S, a photo‐thermoperiod‐sensitive genic male sterile (PTGMS) line famous for high combining ability and currently widely used in central China.

### GCA and SCA contribute variously to heterosis and agronomic traits in hybrids

We determined the effects of male parents, female parents, female parents×male parents and environments of yield‐related traits of the hybrid population by two‐factor variance analysis (see Methods). Male parents, female parents and female parents × male parents were significant for all traits (Table 1). According to quantitative genetics theory (Allard, 1999; Bulmer, 1980), we calculated the additive genetic variances for male ($\delta_{m}^{2}$) and female ($\delta_{f}^{2}$) parents, the nonadditive genetic variance for female parents × male parents ($\delta_{\mathrm{mf}}^{2}$), environmental variance ($\delta_{e}^{2}$), and narrow‐sense (*h* ^*2*^) and broad‐sense (*H* ^*2*^) heritability of all traits assessed in the NCII population. With these results, we found that additive effects (GCA) and nonadditive effects (SCA) contributed in various ways to different agronomic traits. Thus, different traits had different *h* ^*2*^ and *H* ^*2*^. Among all traits, grain length‐to‐width ratio had the highest heritability and grain yield had the lowest.

**Table 1:** Variance and genetic analyses of NCII population. Numbers larger than 100 were rounded to whole numbers

Trait	Males		Females		Males × Females		Replications		$\delta_{f}^{2}$ c	$\delta_{m}^{2}$	$\delta_{\mathrm{mf}}^{2}$	$\delta_{e}^{2}$	*h* ^*2*^	*H* ^*2*^
	MS^a^	Sig.^b^	MS	Sig.	MS	Sig.	MS	Sig.						
Plant height	660	****	3255	****	70.2463	****	35.7881	****	10.8409	43.8992	12.8182	31.7917	0.5526	0.6820
Heading date	1923	****	10226	****	453	****	700	****	33.2070	105	116	105	0.3849	0.7066
Panicle length	19.6137	****	664	****	3.8945	****	38.1437	****	2.2923	1.3099	0.9210	1.1314	0.6370	0.7999
Grain length	1.6917	****	11.7267	****	0.1427	****	0.0690		0.0402	0.1291	0.0390	0.0257	0.7236	0.8903
Grain width	0.1143	****	0.7716	****	0.0134	****	0.0065		0.0026	0.0084	0.0036	0.0027	0.6395	0.8463
Grain length to width	0.5907	****	5.8445	****	0.0464	****	0.0027		0.0201	0.0454	0.0130	0.0075	0.7619	0.9127
Effective panicles	16.9616	****	76.0305	****	11.8453	****	54.9942	****	0.2229	0.4264	3.0700	2.6354	0.1022	0.5853
Flower number/panicle	3853	****	20396	****	1724	****	2293	**	64.8357	177	443	394	0.2245	0.6353
Seed setting rate	7.6780	****	15.6134	****	2.6231	****	4.5191	***	0.0451	0.4212	0.6908	0.5506	0.2731	0.6776
Grain number	591051	****	422185	****	245799	****	3512684	****	612	28771	56793	75421	0.1818	0.5333
Grain weight	27.6836	****	148	***	5.3199	****	7.7654	***	0.4944	1.8636	1.4188	1.0636	0.4872	0.7803
Yield	272	****	881	****	152	****	2354	****	2.5328	10.0488	35.2561	46.1265	0.1339	0.5091

a Component mean squares from variance analysis.

b Significance of *F*‐test, **, *P *<* *0.01; ***, *P *<* *0.001; ****, *P *<* *0.0001.

c Additive genetic variance of male parents ($\delta_{f}^{2}$) and female parents ($\delta_{m}^{2}$), nonadditive genetic variance of male parents × female parents ($\delta_{\mathrm{mf}}^{2}$), environmental variances ($\delta_{w}^{2}$), narrow‐sense heritability (*h* ^*2*^) and broad‐sense heritability (*H* ^*2*^).

To clarify relationships among combining ability, heterotic advantage and phenotype, we calculated the general combining ability (GCA) of male and female parents, specific combining ability (SCA) of individual hybrids and heterotic advantage (HA) of each hybrid (Figure S5a, b). There were high correlations among phenotype, SCA, GCA and heterotic advantage for all traits; phenotype was always positively correlated with GCA and SCA, SCA always positively correlated with heterotic advantage, and GCA was always negatively, or not, correlated with SCA. Among the parameters of hybrid grain yield, SCA was most relevant to phenotype (*r *=* *0.73) and heterotic advantage (*r *=* *0.71; Figure S5a). GCA also showed highly positive correlations with grain yield (*r *=* *0.69), but a low correlation with heterotic advantage (*r *=* *0.21). Therefore, nonadditive genetic effects played a dominant role in heterosis and both additive and nonadditive effects were important to grain yield *per se*. These parameters contributed variously to phenotype and heterotic advantage of different traits (Figure S5a, b). For traits with high heritability such as grain length, GCA contributed more than SCA to phenotype (Figure S5a). Among all 12 traits, mean heterosis of a trait was negatively correlated with *h* ^2^ (*r *=* *−0.71) and *H* ^*2*^ (*r *=* *−0.80; Figure S5c).

### Correlations and distributions of combining ability of agronomic traits

**Figure 2:**
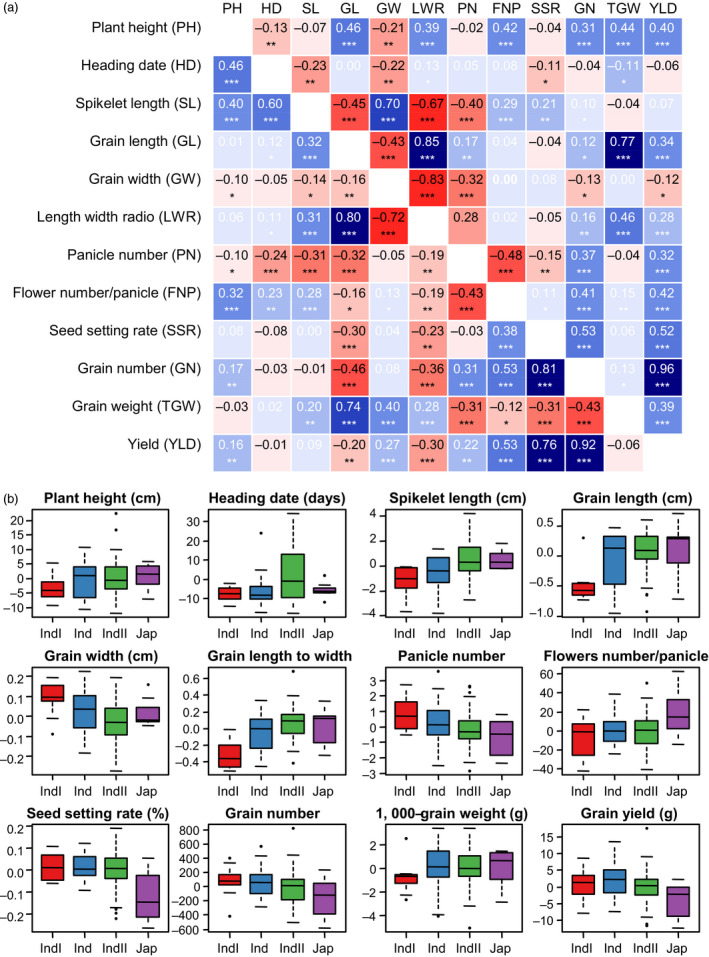
Phenotypic analysis reveals combining ability relationships and subpopulation characteristics. (a) A heatmap depicting Pearson's correlation coefficients between GCAs (lower triangle) and SCAs (upper triangle) for agronomic traits across all varieties within the study. Trait acronyms are in parentheses. Asterisks indicate significant correlations using a two‐tailed *t*‐test (**, *P *<* *0.001; ***, *P *<* *0.0001). (b) Phenotypic distributions of GCA for agronomic traits, divided by the *indicia* (IndI), *indica* intermediate (Ind), *indica II* (IndII) and *japonica* (Jap) subpopulations. The number of varieties within each subpopulation was respectively 19, 23, 46 and 8.

As GCA and SCA contributed variously to different traits, the correlations among GCAs or SCAs can be quite different from that among traits *per se*. The correlations among GCAs of agronomic traits are shown below the diagonal in Figure 2a, and correlations among SCAs are shown above the diagonal. It is clear that the correlations between GCAs of two traits may be quite different from these between SCAs. For example, GCA_Plant height_ was positively correlated with GCA_Heading date_ (*r *=* *0.46, *P *<* *0.001), but SCA_Plant height_ was negatively correlated with SCA_Heading date_ (*r *=* *−0.13, *P *<* *0.01). Since plant height was positively correlated with heading date in the F_1_ population (*r *=* *0.15, *P *<* *0.01; Figure S5), there were both positive additive effects and negative nonadditive effects between the two traits. Thus, dissection of the phenotype into GCA and SCA also helped to explain the genetic relationships among agronomic traits.

Parental GCA for agronomic traits ranged widely and showed distinct distributions within subpopulations (Figure 2b). Parental lines from *indica II* showed an average higher GCA for heading date. Those in *indica I* showed lower GCA for grain length and higher GCA for grain width. Parental lines from *japonica* showed higher GCA for flower number per panicle, and this may have contributed to the high heterosis in *indica–japonica* crosses. However, *japonica* parental lines showed lower GCA for seed setting rate possibly caused by the reproductive isolation between *indica* and *japonica*. It seems that the subpopulation structure explained much of the variance for parental GCA.

### Loci associated with parental GCA

To analyse the genetic architecture of complex agronomic traits and their combining ability, we performed genome‐wide association scans using a **Q + K** model (Yu *et al*., 2006). The GCA of each parent for each trait was considered the phenotype of that parent and SCA of each hybrid. We respectively identified 5, 11, 7 and 12 significant loci for parental agronomic traits, parental GCA, F_1_ agronomic traits and F_1_ SCA (Table S1). Detailed association results for each trait and population are given in Figures S6 and S17 and Table S2.

**Figure 3:**
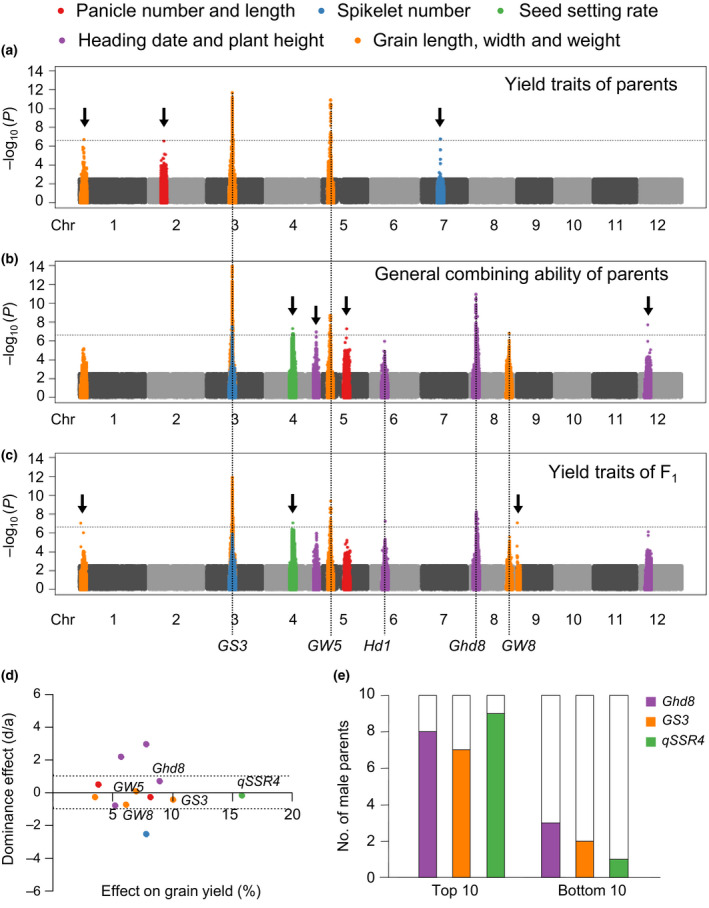
GWAS reveals the genetic basis of GCA. (a‐c) Manhattan plot displaying the GWAS results of parental yield traits (a), parental GCA of yield traits (b) and F_1_ yield traits (c). Negative log_10_ *P* values from linear mixed model (*y*‐axis) are plotted against SNP positions (*x*‐axis) on each of the 12 rice chromosomes. Arrows indicate newly identified loci for yield traits. (d) Plots of the dominance effect (d/a) and the allele effect on grain yield for 12 associations identified in GCA traits. (e) Genotype frequency of three advantage loci in the top 10 and bottom 10 parents of GCA of grain yield.

Significant loci for parental agronomic traits, parental GCA and F_1_ agronomic traits are summarized in Figure 3a–c. By comparing associations in Manhattan plots, we found two grain‐shaped genes, *GS3* and *GW5*, were significant in all three groups. A grain width QTL on chromosome 1, we named *qGW1*, was significant in both parents and F_1_ phenotype but not parental GCA (Figure 3a and Figure S14). Most peaks of parental GCA were consistent with that of F_1_ agronomic traits but not parental agronomic traits (Figure 3a–c). This indicated that the effects of some genes differed between the parental lines and hybrids. Although most loci identified in F_1_ agronomic traits were also identified in parental GCA, they showed higher significance and explained more of the variance for GCA (Table S2). GCA is therefore a good source for studying gene effects in hybrids.

Grain yield is a complicated trait, and no significant loci were identified for this trait or its GCA. As other agronomic traits were significantly correlated with grain yield, we evaluated the effects of loci identified for other traits on grain yield (Figure 3d). Among these loci, two known genes, *Ghd8* and *GS3*, and a QTL for seed setting rate, namely *qSSR4*, contributed most to grain yield. The three loci explained 30.3% of the variance for parental GCA in grain yield and 18.5% of variance for F_1_ grain yield. Comparing the top 10 and bottom 10 parents for GCA of grain yield, it became clear that superior loci were accumulated in parents with high GCA (Figure 3d, e). Therefore, knowing the genetic basis of GCA, we will be able to identify inbred lines with high GCA by molecular markers instead of time‐ and labour‐consuming mating designs.

### Loci contributing to distinct distributions of GCA within subpopulations

**Figure 4:**
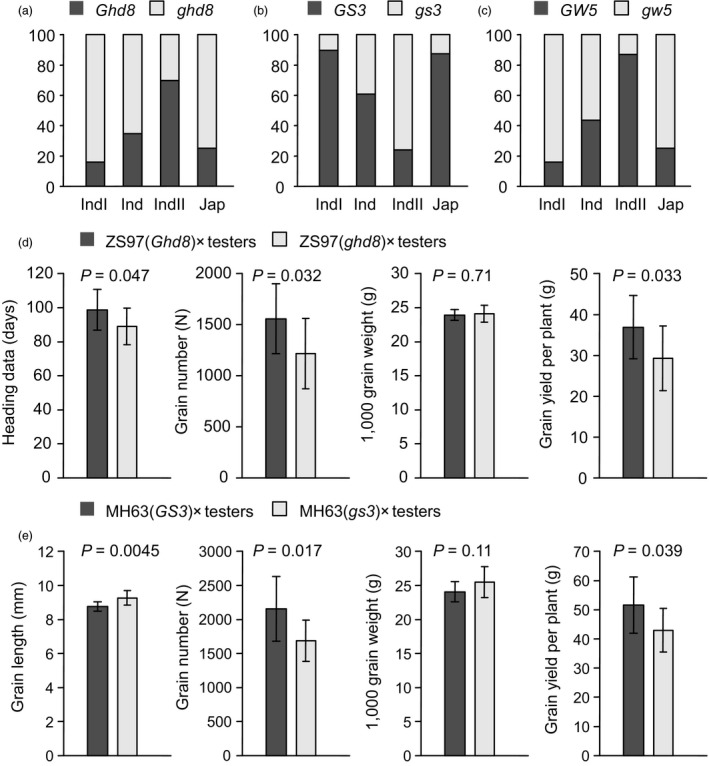
Subpopulation distribution and validation of GCA QTLs. (a‐c) The proportion of different alleles of *Ghd8* (a), *GS3* (b) and *GW5* (c) in rice subpopulations. (d) Heading date, grain number, 1000‐grain weight and grain yield of hybrids from ZS97(*Ghd8*) × testers and ZS97(*ghd8*) × testers. (e) Grain length, grain number, 1000‐grain weight and grain yield of hybrids from MH63(*GS3*) × testers and MH63(*gs3*) × testers.

Population structure (Figure 2b) and GCA QTLs (Figure 3a) both explained large proportions of phenotypic variation in parental GCA. We further linked the variation in these QTLs to population structure and found that the distributions of different alleles of these QTLs were consistent with phenotypic variation in subpopulations. Natural variations in heading date gene *Ghd8*, grain length gene *GS3* and grain width gene *GW5* were examined in previous studies (Lu *et al*., 2013; Zhang *et al*., 2015). The survey of genotypes of the three genes in our GWAS panel indicated that *Ghd8* and *GW5* mainly existed in *indica II*, and *GS3* mainly existed in *indica I* (Figure 4a). Thus, the distinct distributions of these QTLs contributed to the differentiation of parental GCA in the subpopulations.

### Validation of the effects of *GS3* and *Ghd8* on GCA

To evaluate the use of GCA QTLs identified in our study, we performed a further mating design using near‐isogenic lines (NILs) with the QTLs. NILs possessing *GS3* and *Ghd8* were used to validate the GCA effects of the two genes (Figure 4d, e). NILs MH63(*GS3*), MH63(*gs3*), ZS97(*Ghd8*) and ZS97(*ghd8*) were crossed to several male sterile and maintainer lines (Table S3). Hybrids from ZS97(*Ghd8*) × testers showed significantly later heading dates and higher grain yield than hybrids from ZS97(*ghd8*) × testers, indicating that *Ghd8* conferred positive GCA effects for both heading date and grain yield (Figure 4d). Hybrids from MH63(*GS3*) × testers had significantly shorter grain length and higher grain yield than hybrids from MH63(*gs3*) × testers (Figure 4e). Thus, *GS3* had a negative GCA effect on grain length but positive GCA effect on grain yield. These results were consistent with GWAS of GCA and revealed that complex GCA effects in grain yield can be partitioned into genes with GCA effects.

### Identification of SCA QTLs for agronomic traits

Due to dominant effects for SCA, an additive model is not suitable for identifying associations of SCA effects (Figures S6‐S17d). In order to dissect the genetic architecture of SCA, we applied a dominant model according to Huang *et al*. (2015) (See Methods). Using this model, we detected many significant associations for F_1_ SCA that differed from associations involving F_1_ agronomic traits (Figure 5a and Figures S6‐S17e).

**Figure 5:**
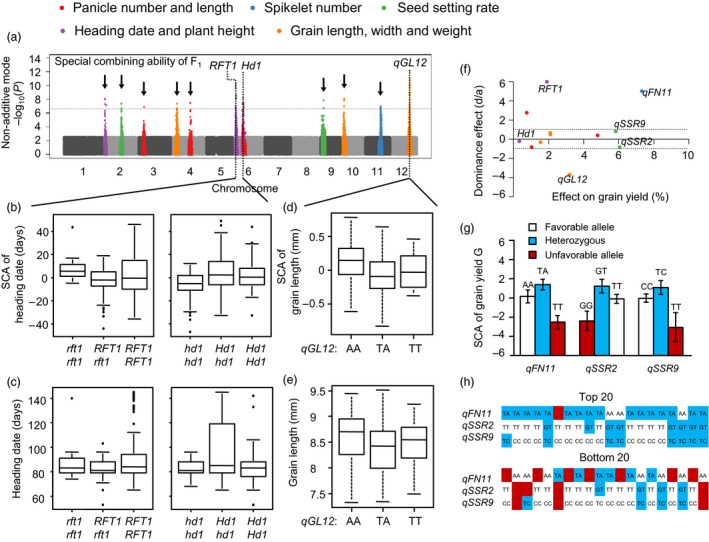
GWAS reveals the genetic basis of SCA. (a) Manhattan plot displaying the GWAS results of SCA using a pseudo‐non‐additive model. Negative log_10_ *P* values from linear mixed model (*y*‐axis) are plotted against SNP positions (*x*‐axis) on each of the 12 rice chromosomes. (b) Interaction between *Hd3a* and *Hd1* led to high SCA of heading date. The genotype combination with high SCA for heading date is coloured brown. (c) Relationships between SCA and trait score for heading date. (d, e) Lead SNP of *qGL12* has an overdominance effect on both SCA (d) and trait score (e) of grain length. (f) Plots of the dominance effect (*d/a*) and the allele effect on grain yield for 12 associations identified in SCA traits. Absolute value of *d/a* > 1 indicates overdominance. (g) Homozygous disadvantage coexisting with heterozygous advantage in three associations for GWAS of SCA. (h) Frequency of advantageous and disadvantageous genotypes in the top 20 and bottom 20 hybrid combinations for SCA of grain yield.

Eleven loci for SCA effects are listed in Figure 5a. Among them, *RFT1* and *Hd1*, known genes for heading date, were associated with SCA of heading date in our GWAS. *RFT1* and *Hd1* were reported to promote and delay flowering in long‐day conditions, respectively (Takahashi *et al*., 2009; Zhao *et al*., 2015b). The functional alleles of the two genes show dominance of SCA of heading date (Figure 5b), but their effects on phenotype were lower than on SCA (Figure 5c). A significant SCA locus for grain length, named *qGL12* on chromosome 12, showed dominance effects on grain length. The dominance effects of these loci on SCA were larger than on phenotypes and were easier to detect. As hybrid phenotypes were the sum of GCA and SCA, those SCA effects might be covered by GCA effects.

We also estimated the effects of SCA loci on grain yield. Of 11 loci identified in GWAS, four showed overdominance for grain yield (Figure 5f). Loci *qFN11*,* qSSR2* and *qSSR9* showed dominance in SCA of grain yield and contributed more than 5% of the phenotypic variation in that trait (Figure 5f, g). Hence, the effects of these associations may be masked by GCA effects. Comparing the top 20 and bottom 20 hybrids for SCA of grain yield, we found that high SCA combinations contained less unfavourable alleles than low SCA combinations (Figure 5h). Thus, high GCA parental lines or high SCA combinations are both caused by pyramided favourable loci. Molecular breeding of these GCA and SCA loci should be effective in hybrid rice breeding.

## Discussion

Combining ability has long been widely used by breeders as an important index of hybrid vigour, but its genetic basis was largely unknown (Lee and Tollenaar, 2007). Clarifying the genetic bases of GCA and SCA is important for molecular breeding of hybrid rice. Selections of parental lines with high GCA and hybrid combinations with high SCA were made by testcrossing in traditional breeding. QTLs for GCA will be useful in identifying elite parent lines. The three GCA QTLs, *Ghd8*,* GS3* and *qSSR4*, explained a large portion of the variation in grain yield in our NCII population (Figure 3). Less than 10% of the parental lines contained all superior alleles of the three QTLs. Molecular marker‐assisted selection (MAS) of those QTLs will help to filter lines lacking one or more of those genes, hence providing the best parental lines with the largest saving in time and labour. QTLs for SCA will also help to select superior hybrids and to avoid inferior ones. Our previous study (Zhou *et al*., 2017) identified *S5* and *qS12* as QTLs for SCA of seed setting rate and grain yield. *S5* is a major locus for *indica*–*japonica* hybrid sterility, and selection of the wide compatibility allele *S5‐n* has been applied in molecular breeding (Chen *et al*., 2008; Yang *et al*., 2012b). In this study, we found *RFT1* and *Hd1* were loci for SCA of heading date and they showed opposite dominance effects on heading date (Figure 5a–c). Thus, MAS of *RFT1* and *Hd1* will also be useful in breeding hybrid combinations with optimal heading dates.

For domestication and artificial selection, the frequencies and distributions of genes may be very different between rice subpopulations, such as *Rc* and *GS3* (Sweeney *et al*., 2007; Takano‐Kai *et al*., 2009). The distinct distributions of GCA QTLs, such as *Ghd8*,* GS3* and *GW5*, contributed to the differentiation of parental GCA in subpopulations (Figures 2b and 4a). This indicates that these genes might be selected in domestication or in breeding. Previous studies showed that the heterosis groups, *indica I* and *indica II*, had different selection histories in 200 regions spanning the rice genome (Xie *et al*., 2015). In the process of hybrid rice breeding, breeders tend to select inbred lines with high GCA, hence selection of these genes.

Low heritability of hybrid rice yield is caused by complex components including environmental sensitivity but can also be caused by long‐term selection for yield that fixes the additive genetic variation (Figure S5c). According to the breeder's equation (Kelly, 1999) *R = ih* ^*2*^ *s* (where *R* is the advance in one generation of selection, *h* ^*2*^ is the heritability, *s* is phenotypic standard deviation of the parental population, and *i* is the intensity of selection), response to selection of a trait is positively correlated with its heritability. Repeated selection tends to fix favourable additive genes, resulting in a decline in both heritability and phenotypic standard deviation. Once genes have been fixed, there will be no further response to selection. With ongoing generations of selective breeding, increasing numbers of additive loci become fixed, the additive genetic variance becomes less and heritability is reduced. As a result, the lower ratio of additive to nonadditive genetic variance leads to a lower efficiency of selection for GCA and a higher requirement for selection of SCA if hybrids are to continue to be superior to conventional inbred cultivars. Ultimately, heterosis will be determined by SCA. Thus, clarifying the genetic basis of SCA becomes increasingly important for hybrid breeding.

These novel insights into the genetic basis of, and relations between, combining ability and hybrid performance will be useful in future work to improve parental lines and create superior hybrids in rice. Combining ability in other crops, such as maize, may have a similar genetic basis to that in rice, but further investigations through large‐scale mating design approaches should be undertaken. We believe a better understanding of combining ability in crops will help in the development of new strategies for breeding superior hybrids and in fulfilling global food security.

## Materials and methods

### Development of the NCII population and genome sequencing

The 96 male parental lines were from a worldwide collection of 529 cultivated rice accessions sequenced in a previous study (Chen *et al*., 2014). All accessions were grown at Wuhan (N 30.49°, E 114.36°) during May to October 2013. The 96 male parents, all with medium heading dates (80–120 days), were crossed with each of the four female parents to obtain at least 100 hybrid seeds for each of the 384 possible hybrids. The female parental lines were photo‐thermo‐sensitive male sterile lines commonly used in hybrid rice production. The four female parents were sequenced on an Illumina HiSeq2000 at 12‐fold genome coverage, generating 96‐bp paired‐end reads.

### Genotyping and population structure analysis

The paired‐end reads of the female parental lines were aligned against the rice reference genome (IRGSP 6.1) using BWA software (Li and Durbin, 2009). SNPs were identified using SAMtools and BCFtools. Only alignments with mapping quality ≥ 40 were used, and bases with base quality ≥ 10 were used to identify SNPs (with parameters of ‐C50, ‐Q10 and ‐q40 for the mpileup subcommand of SAMtools). Only the reads uniquely mapped to the genome sequence were retained for further analysis. Genotypes of 1483 varieties identified in a previous study (Zhao *et al*., 2015a) were used to impute missing genotypes of the female parents using an LD‐KNN algorithm (Chen *et al*., 2014). We selected SNPs with MAF ≥ 0.05 and missing rate <15% among the 100 parental lines, resulting in 1 663 267 SNPs for further study.

A matrix of pairwise genetic distances derived from simple SNP matching coefficients was used to construct phylogenetic trees using the software PLINK (version 1.9; Purcell *et al*., 2007). MEGA6 software was used to visualize the phylogenetic trees (Tamura *et al*., 2013). The Bayesian clustering program *fastStructure* (Raj *et al*., 2014) was used to calculate varying levels of *K* (*K *=* *1–10). Figures S1a and S2a were generated using the subcommand ditstruct.py in *fastStructure*. The principal component analysis was conducted using the svd() function in R (Ihaka and Gentleman, 1996; version 3.2.2), calculated using SNPs present in all accessions. Genome‐wide LD was estimated using pairwise *r* ^*2*^ between SNPs and calculated using the –r2 –ld‐window‐kb 1000 –ld‐window 99999 –ld‐window‐r2 0 command in PLINK (Purcell *et al*., 2007; version 1.9).

### Phenotyping and statistical analyses

Three replications of the parents and F_1_ populations were grown in the normal rice‐growing season at three locations at Ezhou in Wuhan. Seeds were planted in a seedbed in mid‐May 2014, and approximately 30 plants of each variety/hybrid were transplanted to the field in mid‐June. The planting density was 16.5 cm between plants in rows spaced 26 cm apart. Field management, including irrigation, fertilizer application and pest control, followed normal agricultural practices. Accessions were grown in a randomized block design, and the grains were harvested when fully ripe. Heading date, plant height, panicle length and panicle number were recorded in the field and grain‐related traits, flower number, seed setting rate, grain number, grain length, grain width and grain weight, and were measured utilizing a high‐throughput phenotyping facility (Yang *et al*., 2014). Phenotypic correlations of 12 yield‐related traits were calculated using mean phenotype values. Best linear unbiased predictors (BLUPs) for genetic values were calculated using the mixed.solve() function in the R package rrBLUP (version 4.4). *P*‐values for phenotypic and genetic correlation coefficients were calculated by two‐sided *t*‐tests using the cor.test() function in R (Ihaka and Gentleman, 1996). As the female parents were always sterile, their yield traits could not be measured. In this case, heterotic advantage was defined as the percentage difference between hybrid and the corresponding male parents (Huang *et al*., 2016).

### Analysis of combining ability

As the NCII design is a two‐factor variance design, the effects of male parents, female parents, male parents × female parents and environment were calculated using variance analysis as outlined in the following table.

**Table:** 

Source of variation	df	Mean square	Expected mean square
Replications	*r* − 1		
Males	*m* − 1	MS1	$\delta_{w}^{2}+r\delta_{\mathrm{mf}}^{2}+rf\delta_{m}^{2}$
Females	*f* − 1	MS2	$\delta_{w}^{2}+r\delta_{\mathrm{mf}}^{2}+rm\delta_{f}^{2}$
Males × females	(*m* − 1)(*f* − 1)	MS3	$\delta_{w}^{2}+r\delta_{\mathrm{mf}}^{2}$
Error	(*r* − 1)(*mf* − 1)	MS5	*δ* ^*2*^
Total	*rmf* − 1		

*m*, number of female parents; *f*, number of male parents; *r*, number of replications.

We calculated the additive genetic variance of male parents ($\delta_{f}^{2}$) and female parents ($\delta_{m}^{2}$), nonadditive genetic variance of male parents × female parents ($\delta_{\mathrm{mf}}^{2}$), genetic variance of F_1_ ($\delta_{G}^{2}$), environmental variance ($\delta_{w}^{2}$), phenotypic variance of F_1_ ($\delta_{P}^{2}$), narrow‐sense heritability (*h* ^*2*^) and broad‐sense heritability (*H* ^*2*^). These parameters were calculated by the equations: $\delta_{f}^{2}=\left(\text{MS}2-\text{MS}3\right)/rm;\delta_{m}^{2}=\left(\text{MS}1-\text{MS}3\right)/rf;\delta_{\mathrm{mf}}^{2}=\left(\text{MS}3-\text{MS}4\right)/r;\delta_{w}^{2}=\text{MS}4;\delta_{G}^{2}=\delta_{f}^{2}+\delta_{m}^{2}+\delta_{\mathrm{mf}}^{2};\delta_{P}^{2}=\delta_{f}^{2}+\delta_{m}^{2}+\delta_{\mathrm{mf}}^{2}+\delta_{w}^{2};h^{2}=\delta_{G}^{2}/\delta_{P}^{2};\;\text{and}\;H^{2}=\left(\delta_{f}^{2}+\delta_{m}^{2}\right)/\delta_{P}^{2}$

The mathematical representation of the relationship between phenotype and combining ability for each cross was as follows: Y_*ij*_ = Y + G_*i*_ + G_*j* _+ S_*ij,*_ where Y_*ij*_ was the phenotype value of the hybrid derived from the *i*th male parent and *j*th female parent, Y was the mean phenotype value of all hybrids, G_*i*_ is the general combining ability (GCA) of the *i* th male parent, G_*j*_ was the GCA of the *j*th female parent, and S_*ij*_ was the specific combining ability (SCA) of the hybrid derived from the *i*th male parent and the *j*th female parent. As Y_*i*_ is the mean phenotype of the hybrid derived from the *i*th male parent and Y_*j*_ is the mean phenotype of the hybrid derived from the *j* th female parent, the combining ability was calculated by the following equations:

$$G_{i}=Y_{i}-Y;G_{j}=Y_{j}-Y;S_{\mathrm{ij}}=Y_{\mathrm{ij}}-Y_{i}-Y_{j}-Y$$

### Genome‐wide association analysis

We used 1 663 267 SNPs with minor allele frequencies ≥ 0.05 and missing data rate <15% among the parent lines to carry out GWAS. Population structure (**Q**) was corrected using the first five principal components as fixed effects in our model. Relatedness was estimated using the relative kinship matrix (**K**) of the proportion of shared SNP alleles as a variance–covariance matrix for random genotypic effects. GWAS was performed using a linear mixed model in the EMMAX program (Zhou and Stephens, 2012). We first conducted separate GWAS for yield‐related traits of the parents and NCII population. We took the GCA of each parent as a new trait of the parents and the SCA of each hybrid combination as a new trait of the hybrid. We performed GWAS separately for GCA and SCA using the parental and hybrid genotypes. GWAS results for GCA were normal and consistent with the hybrid yield traits, but SCA effects showed only low levels of significance (>10^−3^). As SCA is due to nonadditive gene action, it could not be explained by additive genotypes. In order to find associations between genotype and SCA, dominant and recessive models were also used. The EMMAX software package simply follows the encoding scheme of the genotype data in the additive model by default. For dominant and recessive models, heterozygous genotypes (ra) were changed to be ‘homozygous genotypes of both the reference alleles' (rr) or ‘homozygous genotypes of both the alternative alleles' (aa) for all the SNPs. The newly made genotype data were then imputed into the EMMAX software package for GWAS. Genome‐wide significance thresholds of the GWAS were determined using a modified Bonferroni correction as described (Li *et al*., 2012) in which the total number of SNPs (M) for threshold calculation was replaced by the effective number of independent SNPs (Me). The calculated genome‐wide significance thresholds, based on a nominal level of 0.05, were *P *=* *2.39 × 10^−7^ for our GWAS scale.

The effect of each association on the corresponding trait was first assessed through a comparison of both homozygous genotypes. We then calculated the average phenotypic values of heterozygous genotypes and homozygous genotypes to estimate an index of dominance effect/additive effect (*d/a*). The ‘*d'* was calculated by the difference between the average phenotypic values of heterozygous genotypes and the population mean. The effect of each association on grain yield was estimated using the *r* ^*2*^ between phenotype and genotype.

## Author contribution statement

H.Z. and J.C. contributed equally to this work. J.C. constructed the experimental population and recorded the phenotypes. W.X. helped to impute the genotypes of parental lines. D.X., G.G, Q.Z., G.W., X.L. and J.X. participated in the field management and logistic work. Y.H. designed and supervised the study. H.Z., J.C. and Y.H. analysed the data and wrote the paper.

## Funding

This work was supported by grants from the Ministry of Science and Technology of China (Grants 2016YFD0100501), National Program on R&D of Transgenic Plants (2016ZX08001002–002), the Natural Science Foundation of China (31821005) and the earmarked fund for the China Agriculture Research System (CARS‐01‐03).

## Conflict of interest

All authors declare that they have no competing interests.

## Ethical standards

All authors declare that this study complies with the current laws of the countries in which the experiments were performed.

## Supporting information


**Figure S1** Genetic architecture of 100 parent lines.
**Figure S2** Genetic architecture of the NCII population.
**Figure S3** Phenotypic correlations and subpopulation characteristics.
**Figure S4** Phenotypic distributions of yield related traits in the NCII population.
**Figure S5** Relations among phenotype, heterosis and combing ability of yield related traits.
**Figure S6** Summary of GWAS results for parental heading date (a), parental GCA of heading date (b), F_1_ heading date (c), F_1_ SCA of heading date (d, e).
**Figure S7** Summary of GWAS results for parental heading date (a), parental GCA of heading date (b), F_1_ heading date (c), F_1_ SCA of heading date (d, e).
**Figure S8** Summary of GWAS results for parental plant height (a), parental GCA of plant height (b), F_1_ plant height (c), F_1_ SCA of plant height (d, e).
**Figure S9** Summary of GWAS results for parental panicle number (a), parental GCA of panicle number (b), F_1_ panicle number (c), F_1_ SCA of panicle number (d, e).
**Figure S10** Summary of GWAS results for parental spikelet number (a), parental GCA of spikelet number (b), F_1_ spikelet number (c), F_1_ SCA of spikelet number (d, e).
**Figure S11** Summary of GWAS results for parental seed setting rate (a), parental GCA of seed setting rate (b), F_1_ seed setting rate (c), F_1_ SCA of seed setting rate (d, e).
**Figure S12** Summary of GWAS results for parental grain number (a), parental GCA of grain number (b), F_1_ grain number (c), F_1_ SCA of grain number (d, e).
**Figure S13** Summary of GWAS results for parental grain length (a), parental GCA of grain length (b), F_1_ grain length (c), F_1_ SCA of grain length (d, e).
**Figure S14** Summary of GWAS results for parental grain width (a), parental GCA of grain width (b), F_1_ grain width (**c**), F_1_ SCA of grain width (d, e).
**Figure S15** Summary of GWAS results for parental grain length to width (a), parental GCA of grain length to width (b), F_1_ grain length to width (c), F_1_ SCA of grain length to width (d, e).
**Figure S16** Summary of GWAS results for parental 1000‐grain weight (**a**), parental GCA of 1000‐grain weight (b), F_1_ 1000‐grain weight (**c**), F_1_ SCA of 1000‐grain weight (d, e).
**Figure S17** Summary of GWAS results for parental grain yield (a), parental GCA of grain yield (b), F_1_ grain yield (c), F_1_ SCA of grain yield (d, e).
**Table S1** Estimated effective number of SNPs and significant thresholds in populations.
**Table S2** Significant genome‐wide associations for parental yield traits, parental GCA, F_1_ yield traits and F_1_ SCA of yield traits.
**Table S3** agronomic traits of hybrids from cross between four NILs and testers.
**Table S4** Phenotype data of parents and F_1_.
