# Supplementary material for: Genome‐wide association analyses reveal the genetic basis of combining ability in rice
Source: Plant Biotechnol J. 2019 Apr 29;17(11):2211–22. doi: 10.1111/pbi.13134 (PMC6790367; doi:10.1111/pbi.13134)
Supplement: Supplementary file 1 — Figure S1 Genetic architecture of 100 parent lines. Figure S2 Genetic architecture of the NCII population. Figure S3 Phenotypic correlations and subpopulation characteristics. Figure S4 Phenotypic distributions of yield related traits in the NCII population. Figure S5 Relations among phenotype, heterosis and combing ability of yield related traits. Figure S6 Summary of GWAS results for parental heading date (a), parental GCA of heading date (b), F1 heading date (c), F1 SCA of heading date (d, e). Figure S7 Summary of GWAS results for parental heading date (a), parental GCA of heading date (b), F1 heading date (c), F1 SCA of heading date (d, e). Figure S8 Summary of GWAS results for parental plant height (a), parental GCA of plant height (b), F1 plant height (c), F1 SCA of plant height (d, e). Figure S9 Summary of GWAS results for parental panicle number (a), parental GCA of panicle number (b), F1 panicle number (c), F1 SCA of panicle number (d, e). Figure S10 Summary of GWAS results for parental spikelet number (a), parental GCA of spikelet number (b), F1 spikelet number (c), F1 SCA of spikelet number (d, e). Figure S11 Summary of GWAS results for parental seed setting rate (a), parental GCA of seed setting rate (b), F1 seed setting rate (c), F1 SCA of seed setting rate (d, e). Figure S12 Summary of GWAS results for parental grain number (a), parental GCA of grain number (b), F1 grain number (c), F1 SCA of grain number (d, e). Figure S13 Summary of GWAS results for parental grain length (a), parental GCA of grain length (b), F1 grain length (c), F1 SCA of grain length (d, e). Figure S14 Summary of GWAS results for parental grain width (a), parental GCA of grain width (b), F1 grain width (c), F1 SCA of grain width (d, e). Figure S15 Summary of GWAS results for parental grain length to width (a), parental GCA of grain length to width (b), F1 grain length to width (c), F1 SCA of grain length to width (d, e). Figure S16 Summary of GWAS results for parental 1000‐grain we [file PBI-17-2211-s001.pdf]

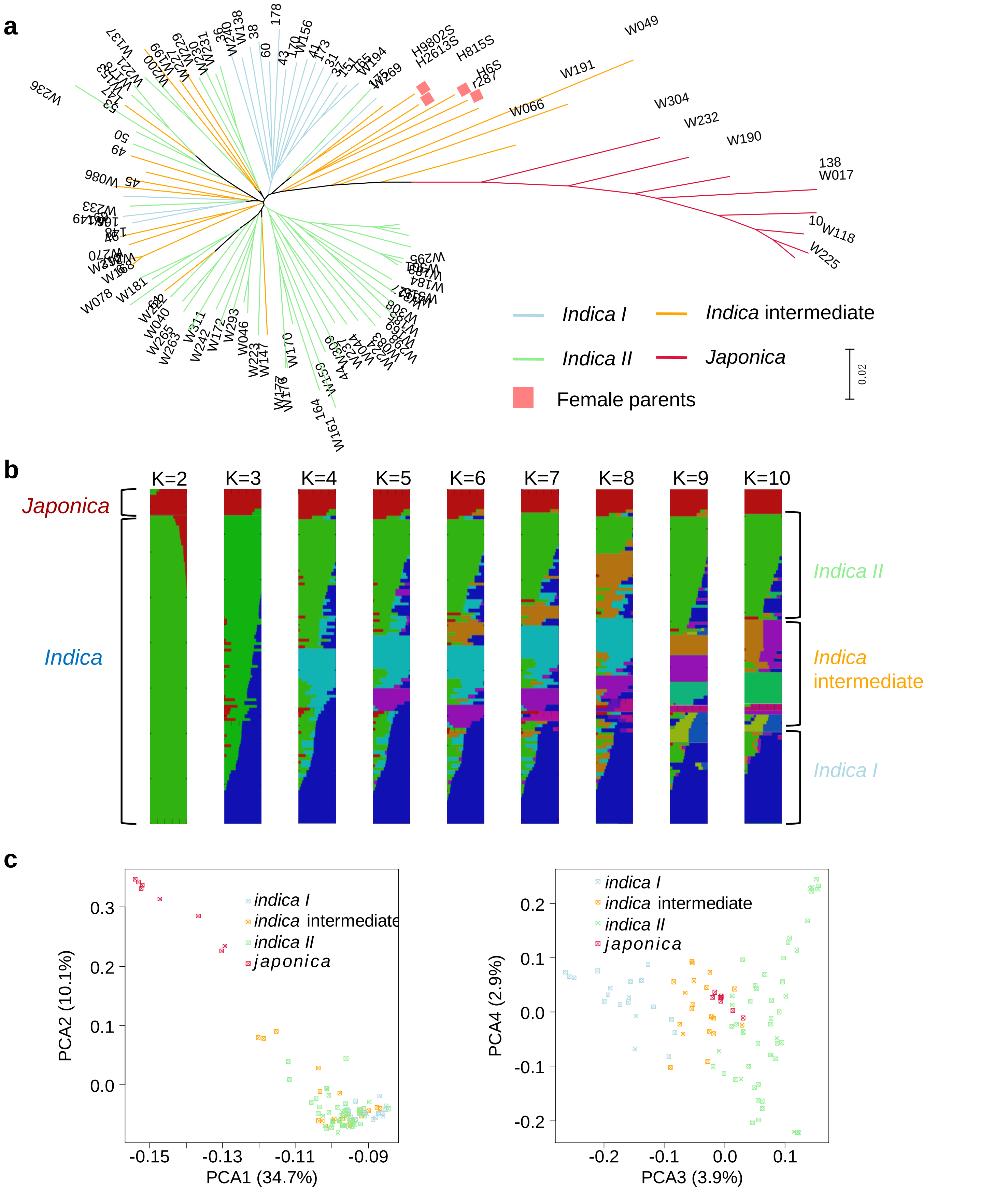

Supplemental Figure 1 Genetic architecture of 100 parent lines. (**a**) Neighbor-joining tree of 96 male parents and 4 female parents used to construct the NCII population. (**b**) Genetic structure of parental lines analyzed using the program *fastStructure*. (**c**) Principle components analysis reveals that the first 4 principle components explained ~50.17% of the genetic variance within parents.

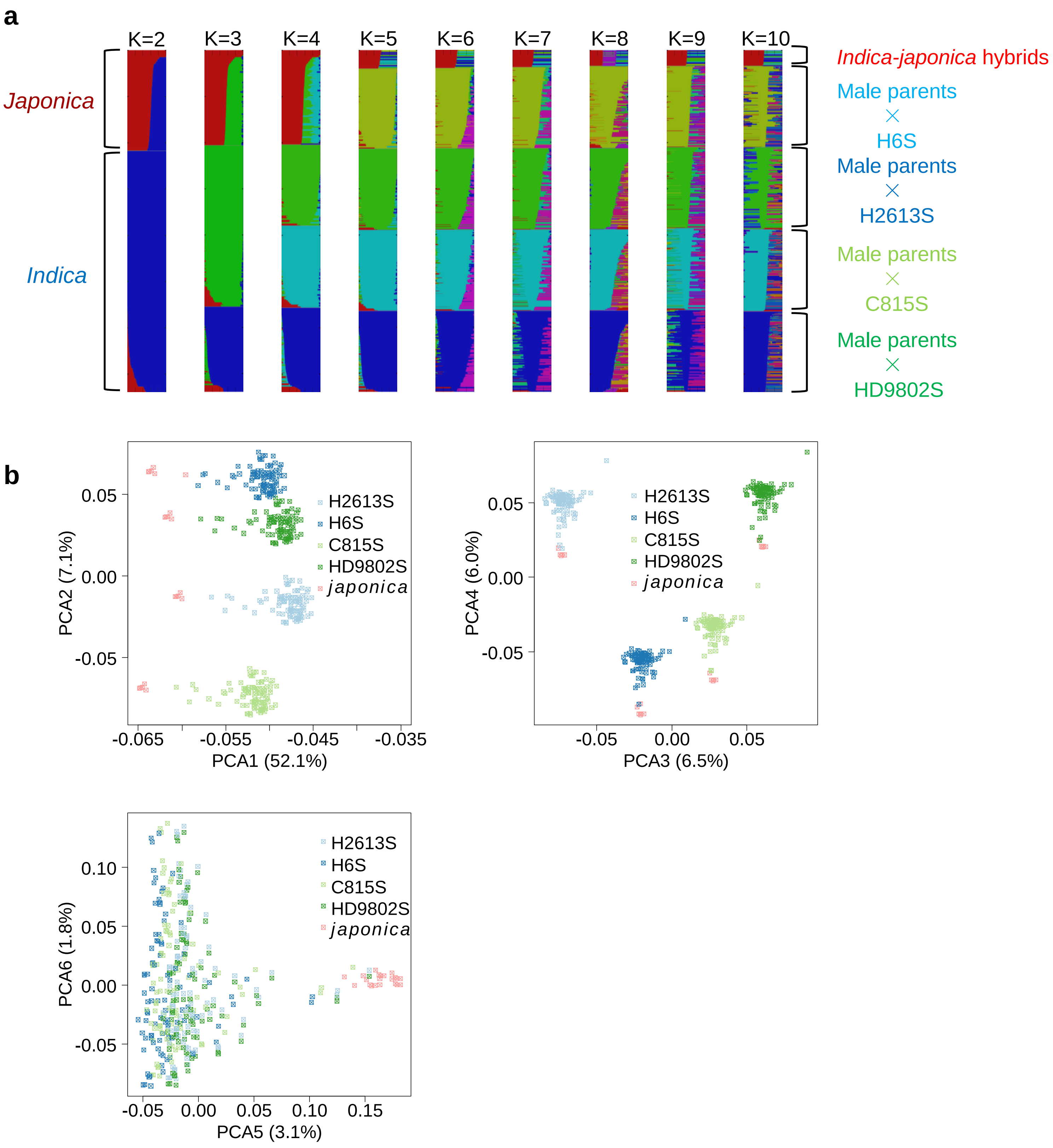

Supplemental Figure 2 Genetic architecture of the NCII population. (**a**) Genetic structure 384 F_1 lines in NCII population analyzed using the program *fastStructure*. (**b**) Principle components analysis reveals that the first 6 principle components explained ~76.6% of the genetic variance within the NCII population.

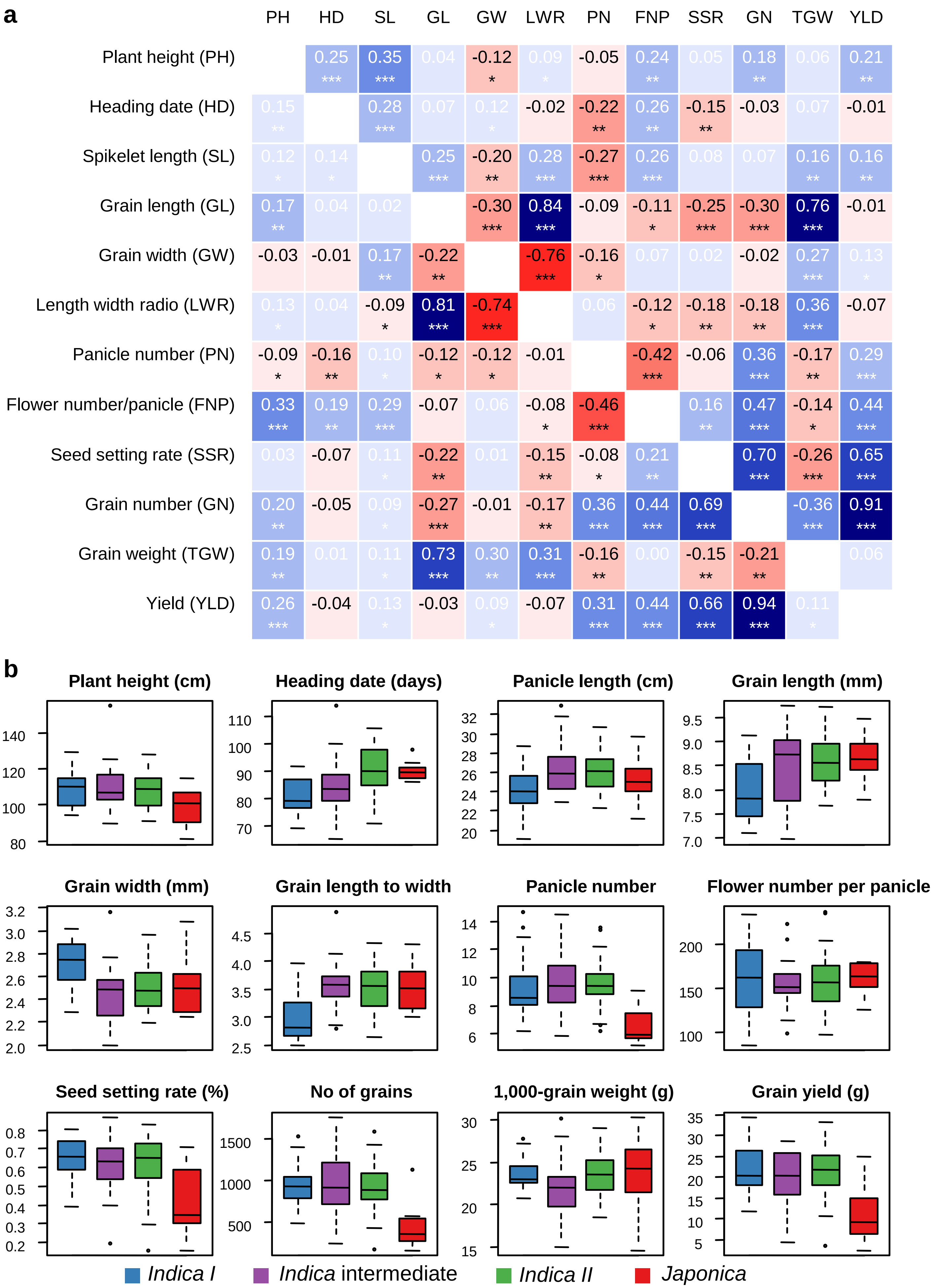

Supplemental Figure 3 Phenotypic correlations and subpopulation characteristics. (**a**) Phenotypic correlations among yield related traits in parental lines (upper triangle) and F_1 generation (lower triangle). (**b**) Phenotypic distributions of yield related traits in the NCII population, divided by the *indica I*, *indica* intermediate, *indica II*, and *japonica* subpopulations. The number of varieties within each subpopulation was respectively 76, 92, 200 and 32.

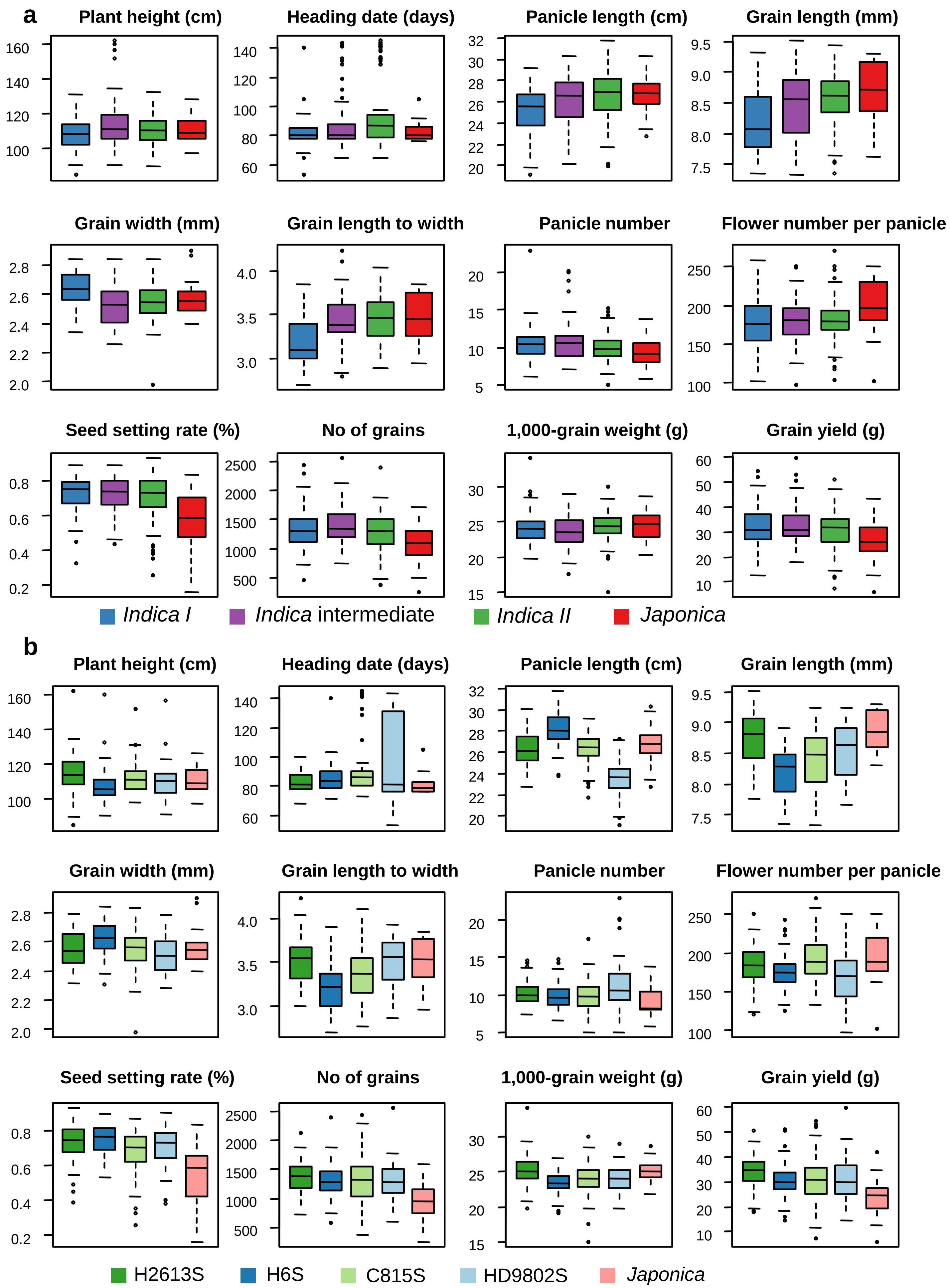

Supplemental Figure 4. Phenotypic distributions of yield related traits in the NCII population, (**a**) Phenotypic divided by the *indica I*, *indica* intermediate, *indica II*, and *japonica* subpopulations. The number of varieties within each subpopulation was respectively 76, 92, 200 and 32. (**b**) Phenotypic distributions

of yield related traits in the NCII population, divided by the four female parent lines and *japonica*

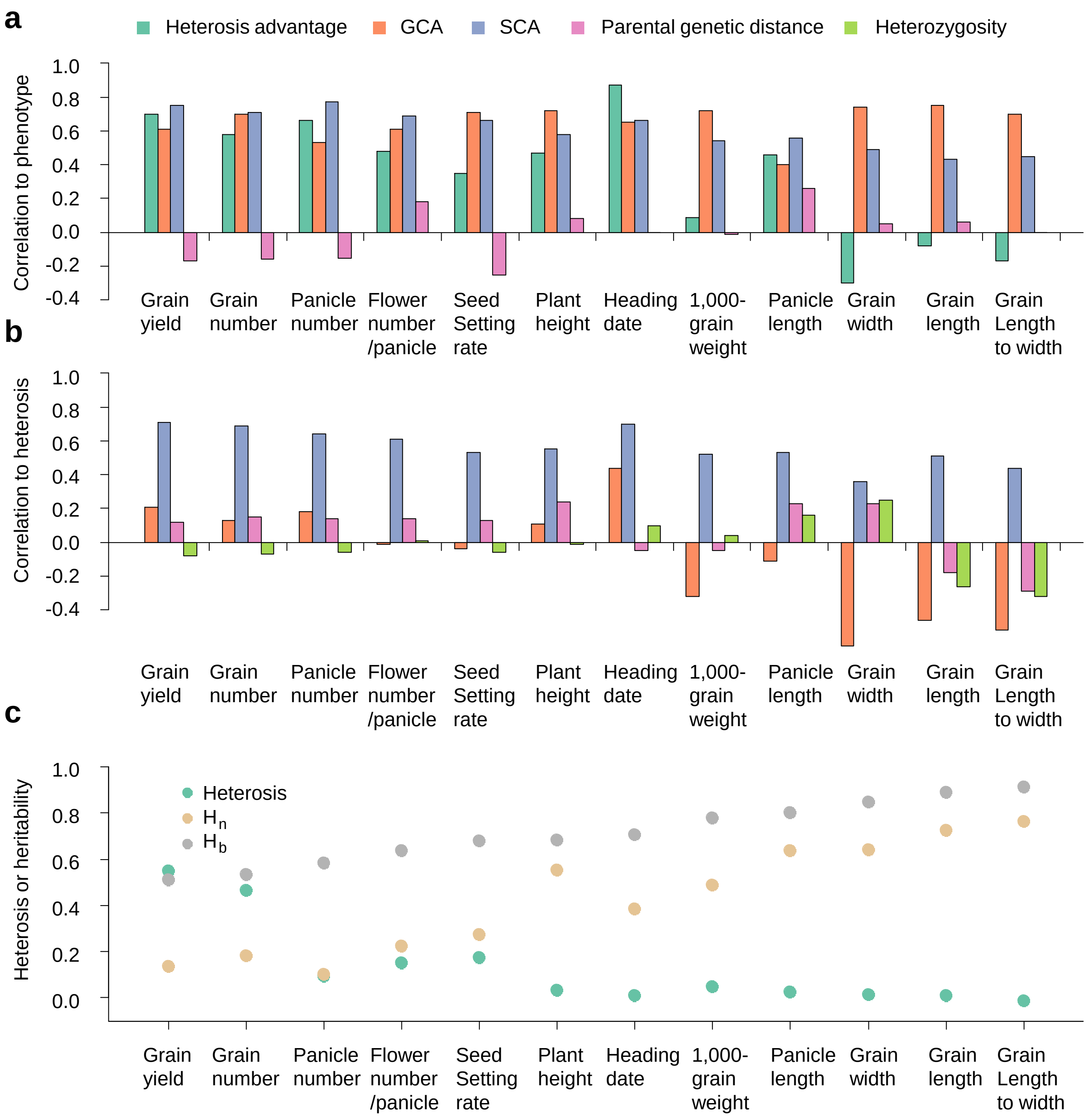

Supplemental Figure 5 Relations among phenotype, heterosis and combing ability of yield related traits. (**a**) The correlation coefficient between phenotype and genetic parameters of yield related traits. (**b**) The correlation coefficient between heterosis and other genetic parameters of yield related traits. (**c**) The relations among mean heterosis, narrow sense heritability (h^2) and broad sense heritability (H^2) of yield related traits.

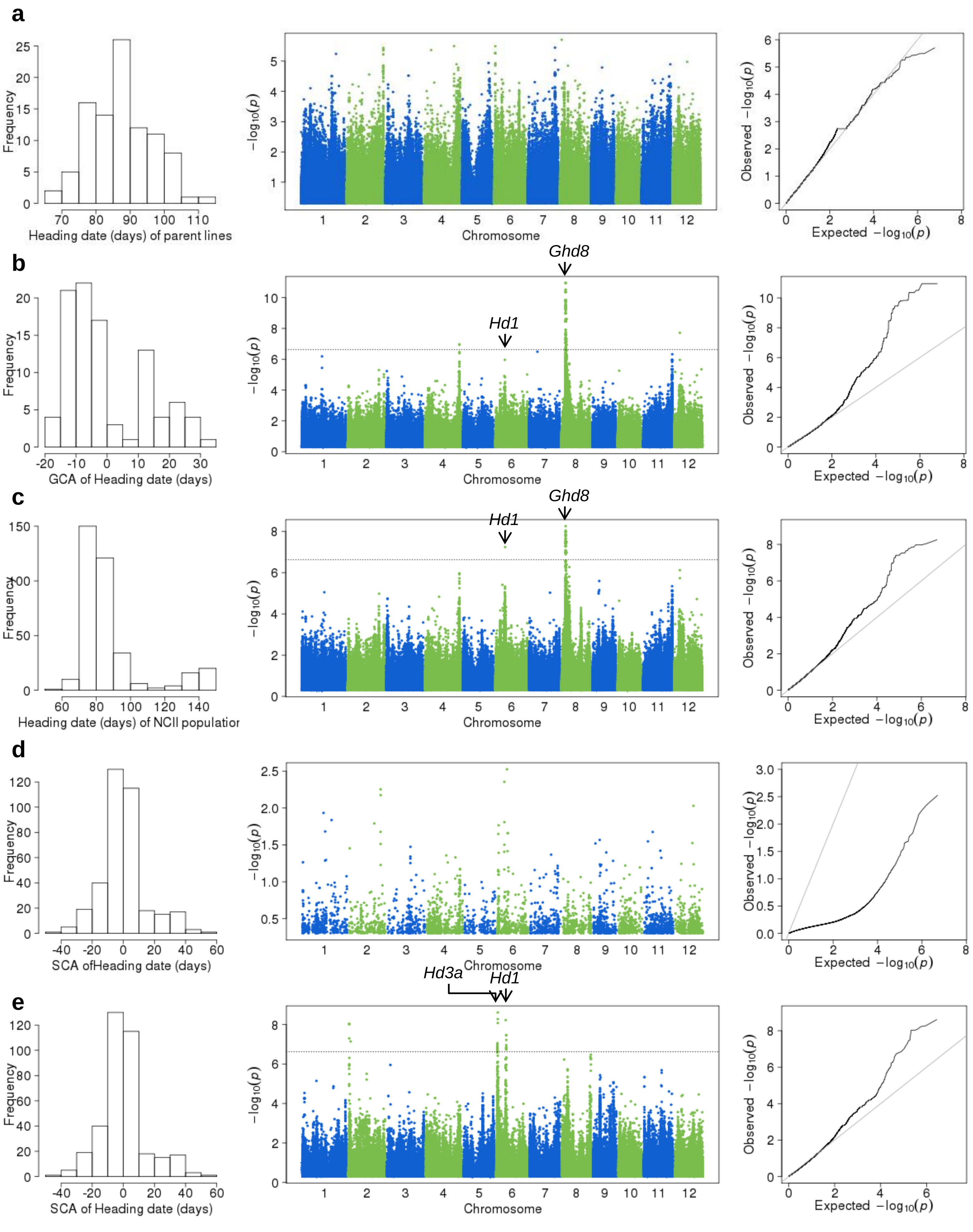

Supplemental Figure 6 Summary of GWAS results for parental heading date (**a**), parental GCA of heading date (**b**), F_1 heading date (**c**), F_1 SCA of heading date (**d, e**). The three frames left to right are phenotypic distribution, Manhattan plots and quantile-quantile (QQ) plots. GWAS in (**a-d**) was based on an additive model and for (**e**) a pseudo non-additive model was used.

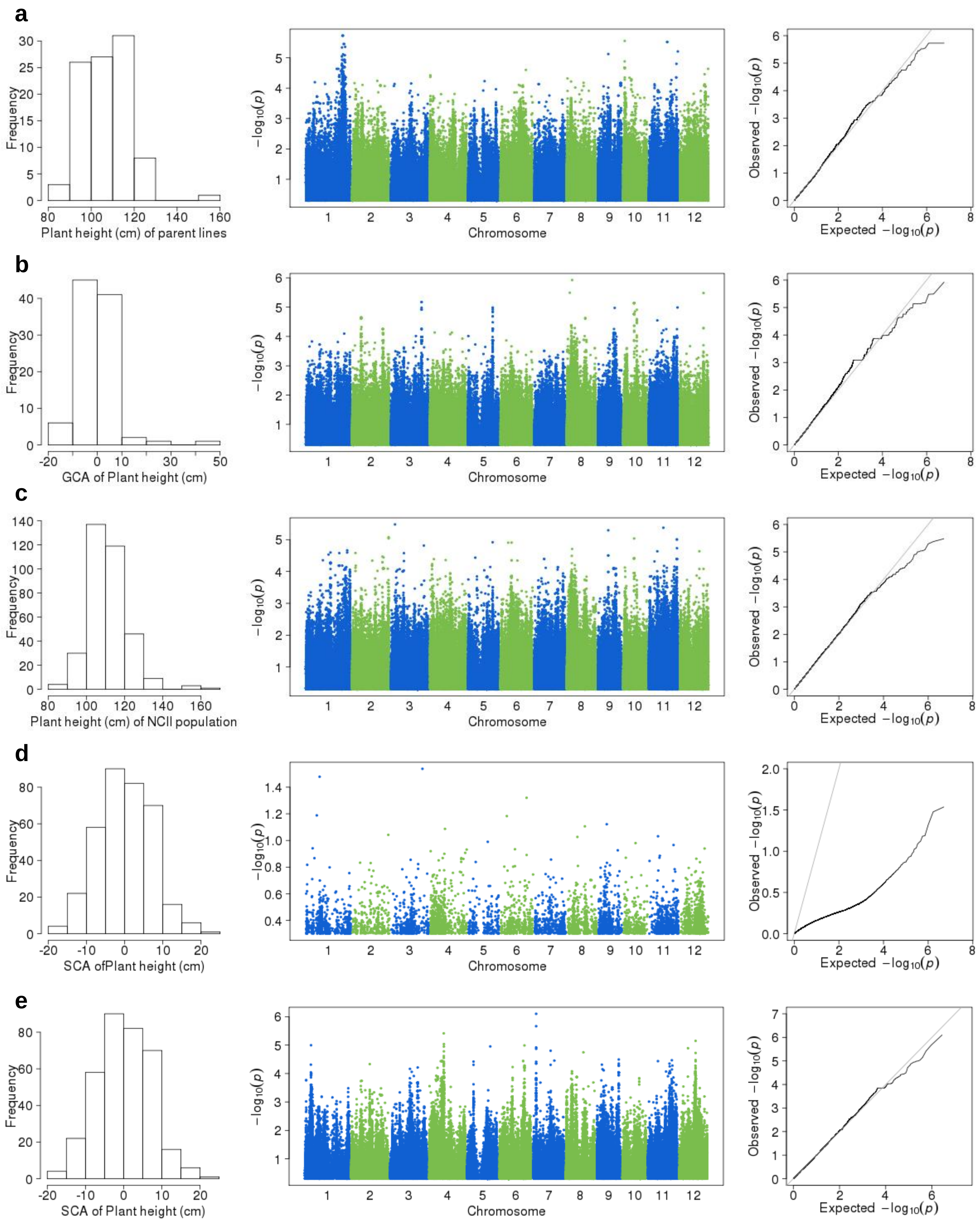

Supplemental Figure 7 Summary of GWAS results for parental heading date (**a**), parental GCA of heading date (**b**), F_1 heading date (**c**), F_1 SCA of heading date (**d, e**). The three frames left to right are phenotypic distribution, Manhattan plots and quantile-quantile (QQ) plots. GWAS in (**a-d**) was based on an additive model and for (**e**) a pseudo non-additive model was used.

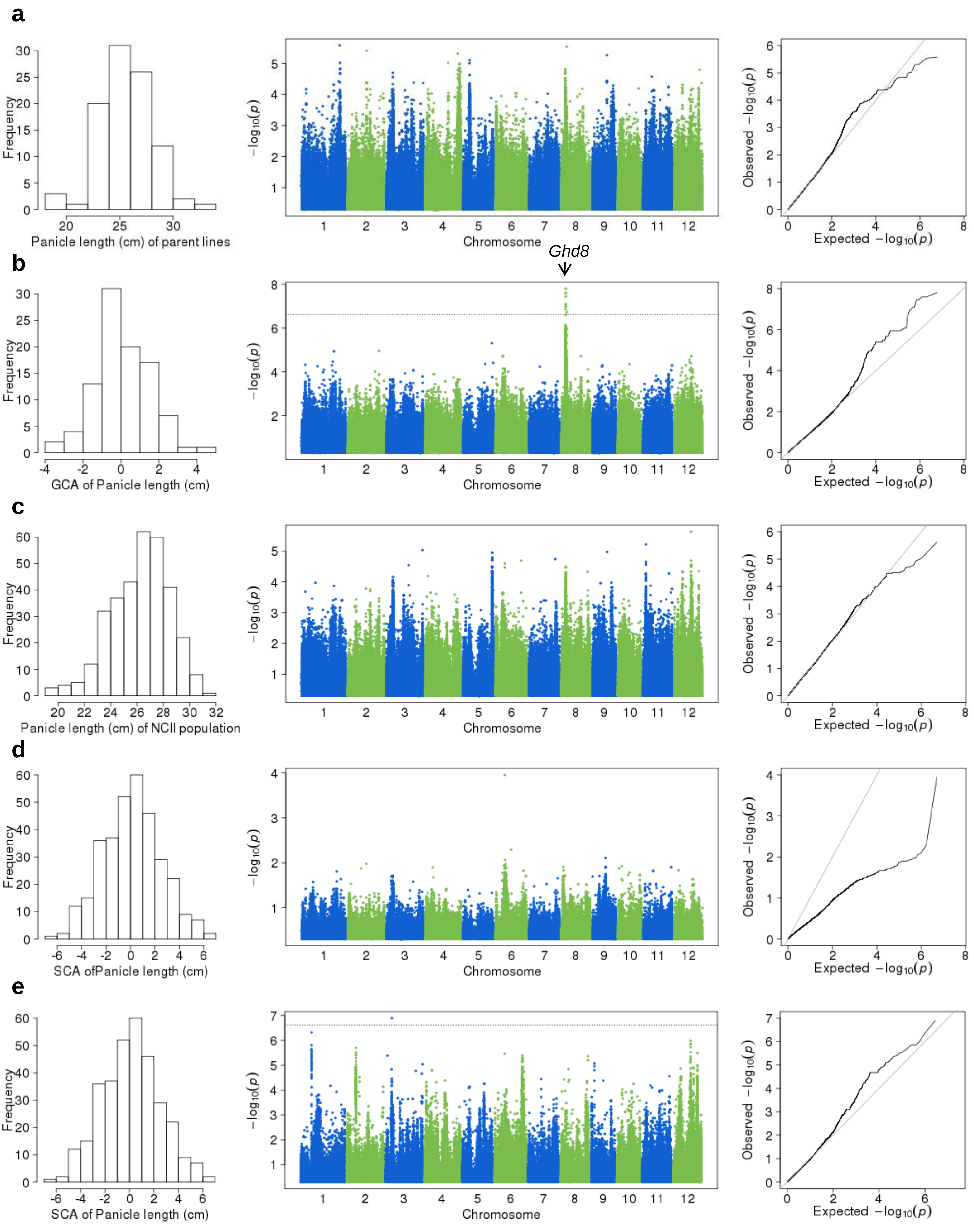

Supplemental Figure 8 Summary of GWAS results for parental plant height (**a**), parental GCA of plant height (**b**), F_1 plant height (**c**), F_1 SCA of plant height (**d, e**). The three frames left to right are phenotypic distribution, Manhattan plots and quantile-quantile (QQ) plots. GWAS in (**a-d**) was based on an additive model and for (**e**) a pseudo non-additive model was used.

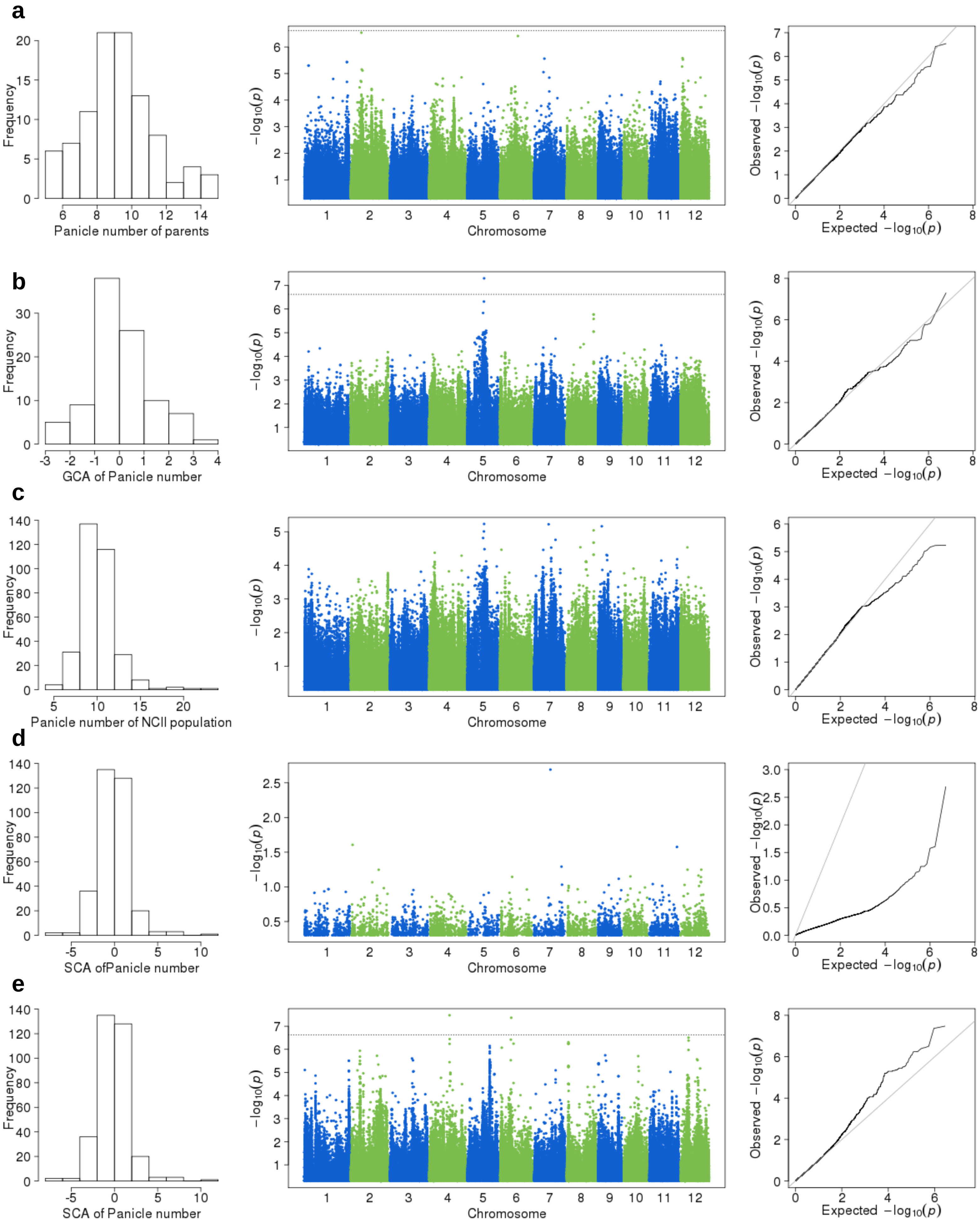

Supplemental Figure 9 Summary of GWAS results for parental panicle number (**a**), parental GCA of panicle number (**b**), F_1 panicle number (**c**), F_1 SCA of panicle number (**d, e**). The three frames left to right are phenotypic distribution, Manhattan plots and quantile-quantile (QQ) plots. GWAS in (**a-d**) was based on an additive model and for (**e**) a pseudo non-additive model was used.

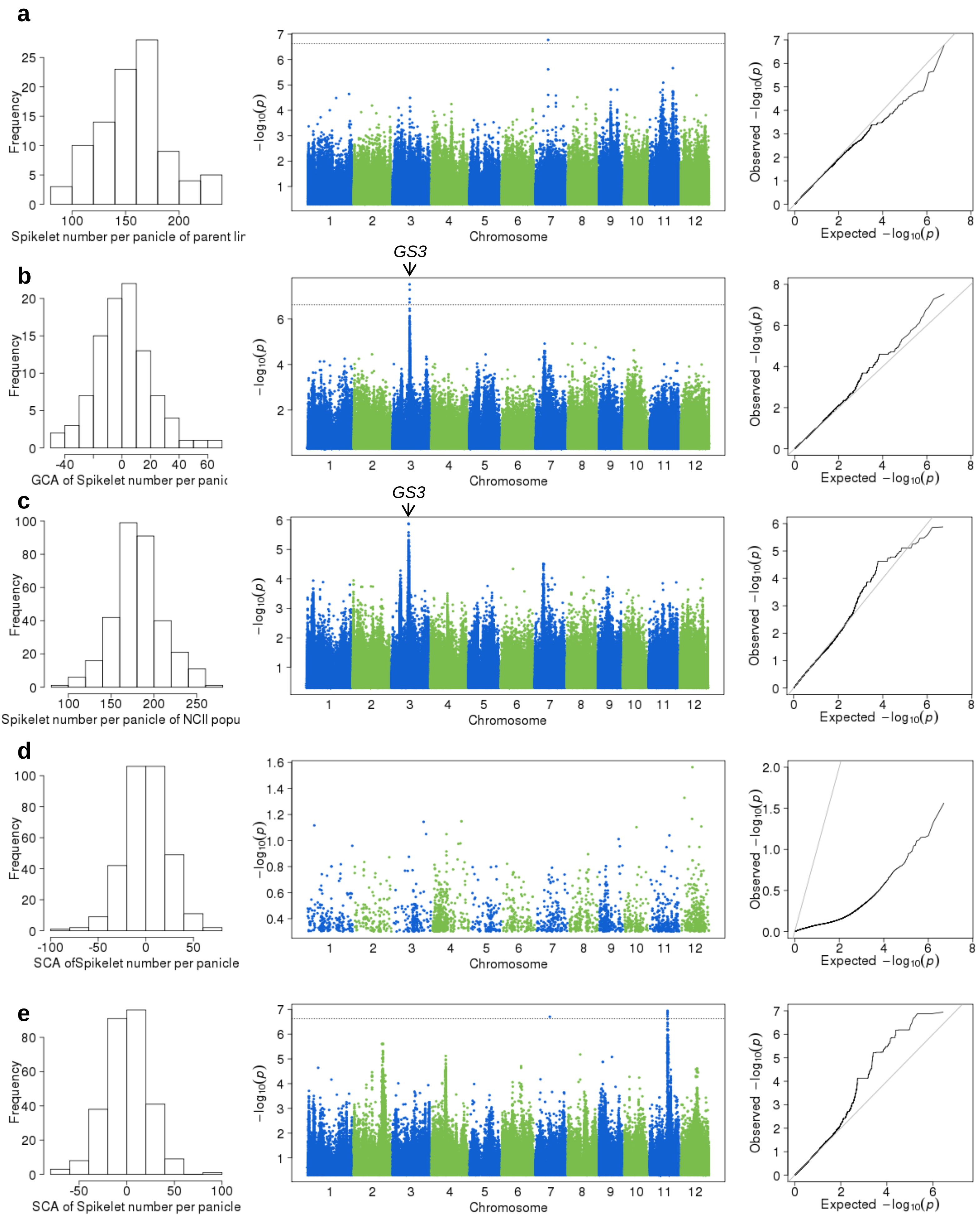

Supplemental Figure 10 Summary of GWAS results for parental spikelet number (**a**), parental GCA of spikelet number (**b**), F_1 spikelet number (**c**), F_1 SCA of spikelet number (**d, e**). The three frames left to right are phenotypic distribution, Manhattan plots and quantile-quantile (QQ) plots. GWAS in (**a-d**) was based on an additive model and for (**e**) a pseudo non-additive model was used.

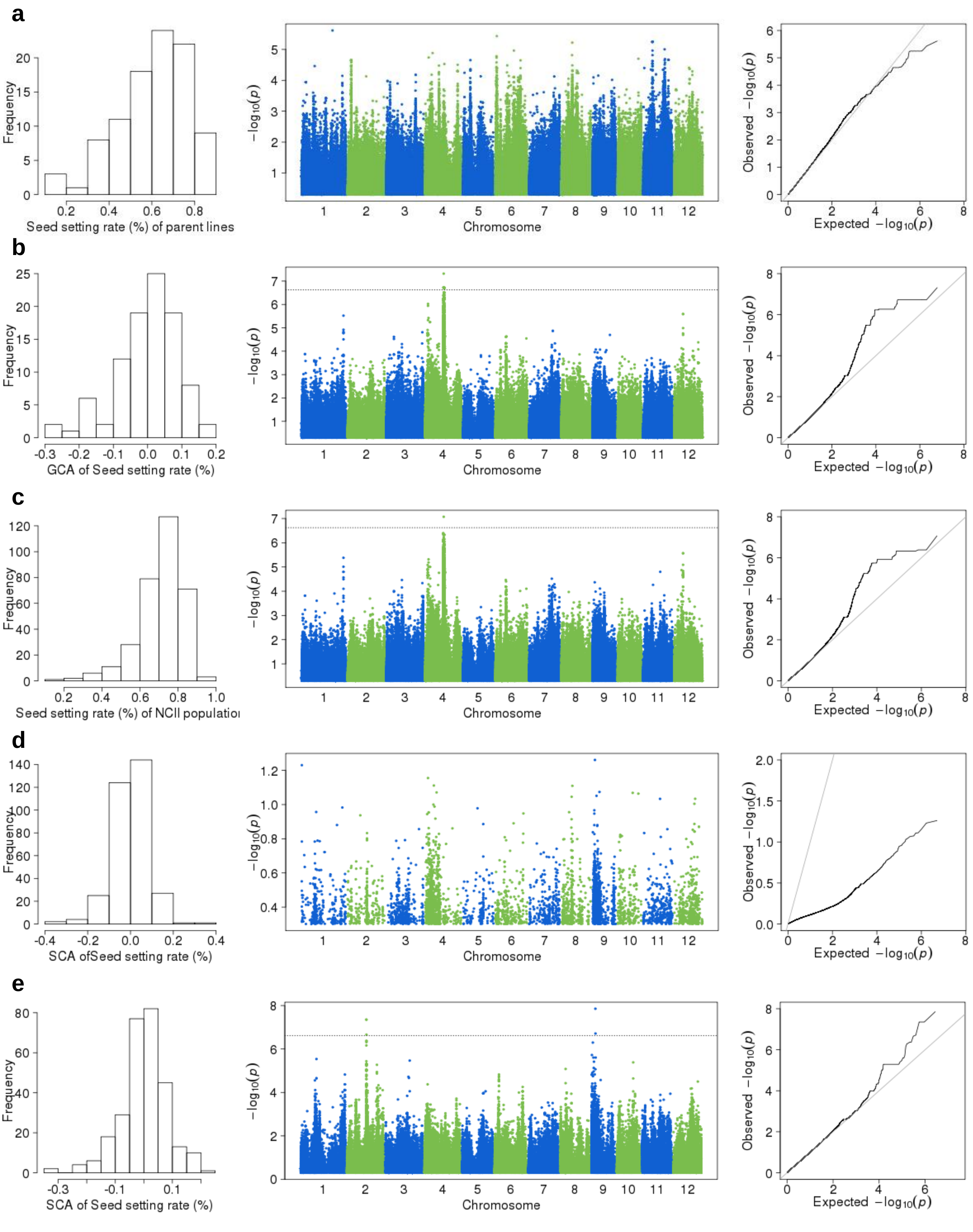

Supplemental Figure 11 Summary of GWAS results for parental seed setting rate (**a**), parental GCA of seed setting rate (**b**), F_1 seed setting rate (**c**), F_1 SCA of seed setting rate (**d, e**). The three frames left to right are phenotypic distribution, Manhattan plots and quantile-quantile (QQ) plots. GWAS in (**a-d**) was based on an additive model and for (**e**) a pseudo non-additive model was used.

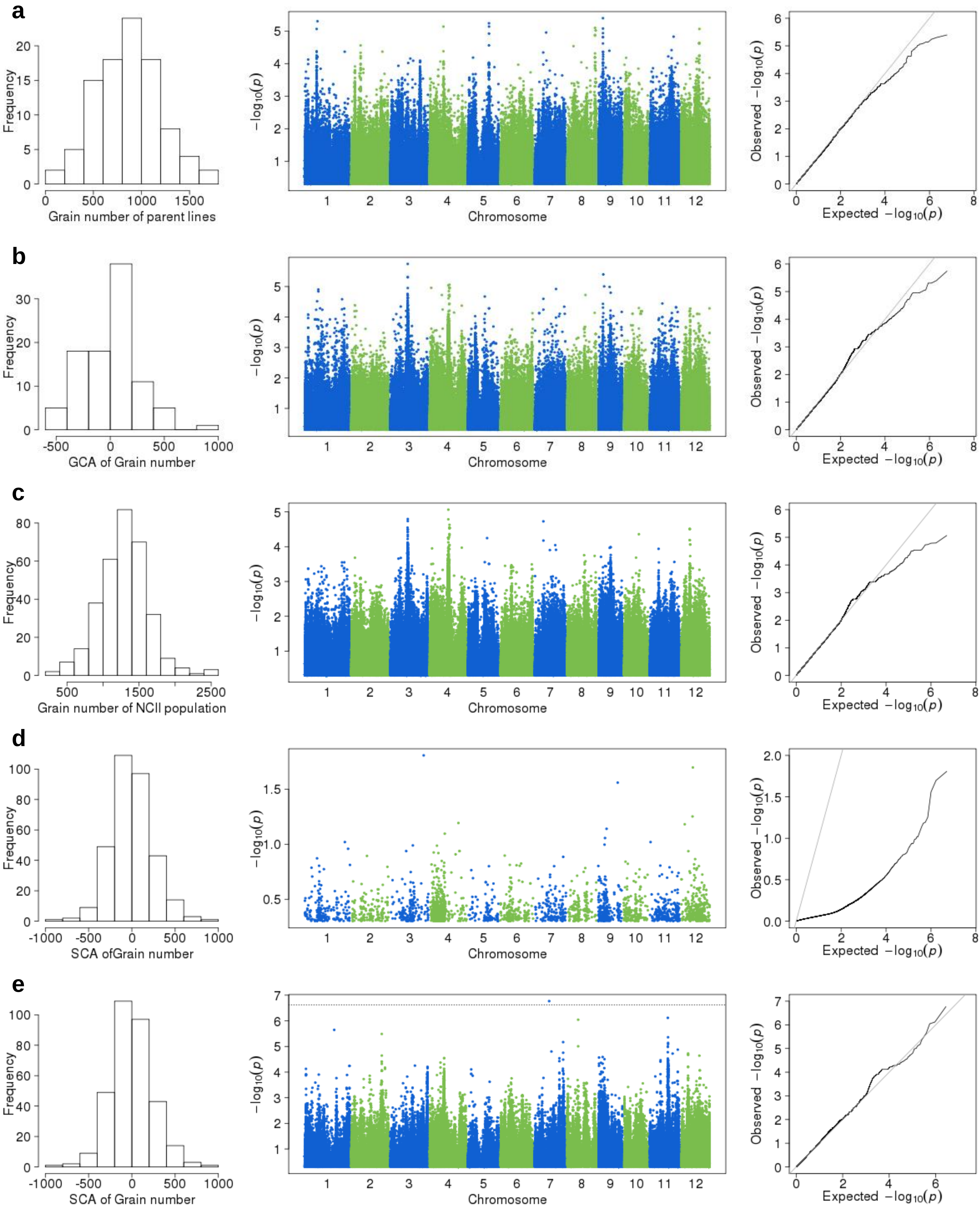

Supplemental Figure 12 Summary of GWAS results for parental grain number (**a**), parental GCA of grain number (**b**), F_1 grain number (**c**), F_1 SCA of grain number (**d, e**). The three frames left to right are phenotypic distribution, Manhattan plots and quantile-quantile (QQ) plots. GWAS in (**a-d**) was based on an additive model and for (**e**) a pseudo non-additive model was used.

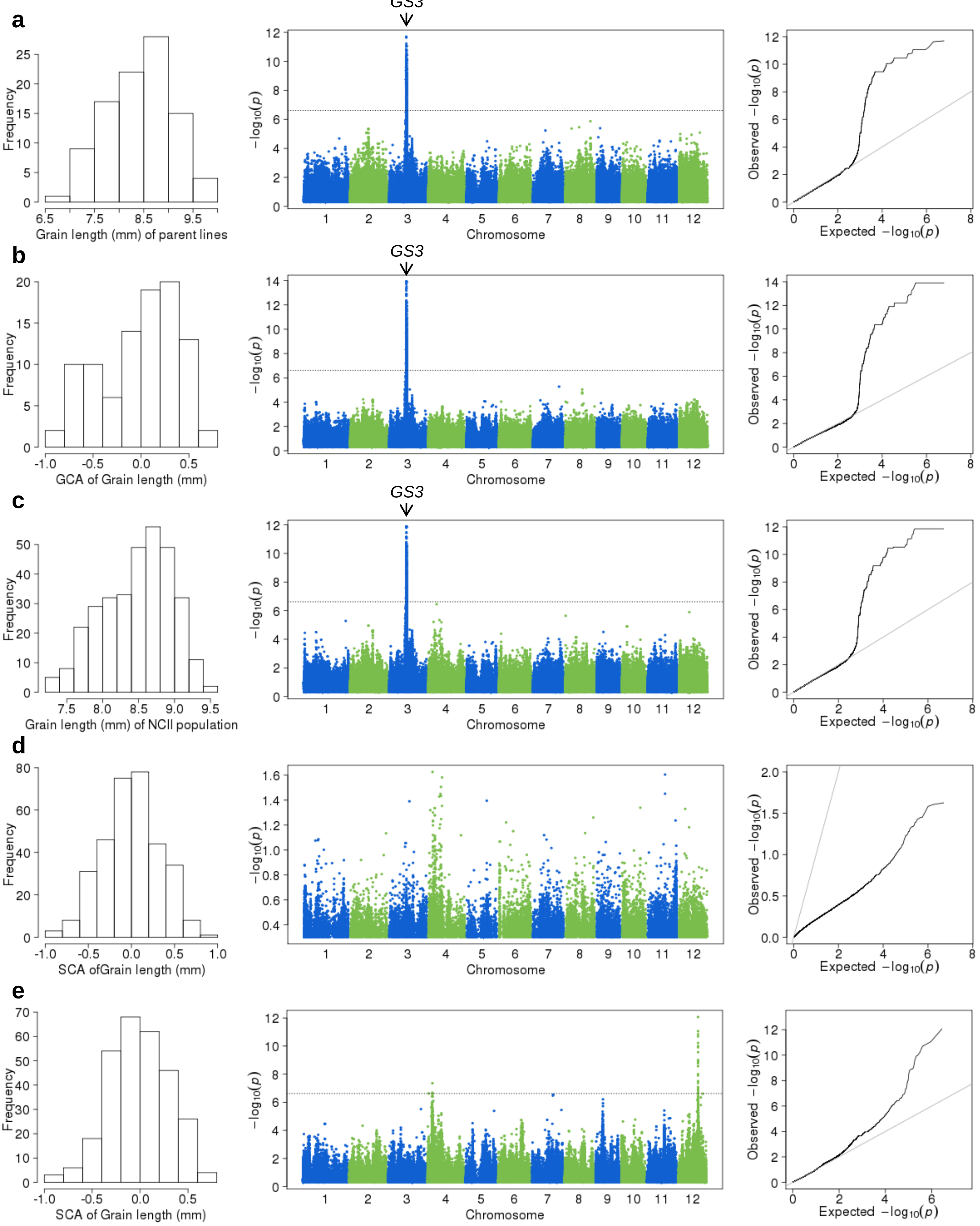

Supplemental Figure 13 Summary of GWAS results for parental grain length (**a**), parental GCA of grain length (**b**), F_1 grain length (**c**), F_1 SCA of grain length (**d, e**). The three frames left to right are phenotypic distribution, Manhattan plots and quantile-quantile (QQ) plots. GWAS in (**a-d**) was based on an additive model and for (**e**) a pseudo non-additive model was used.

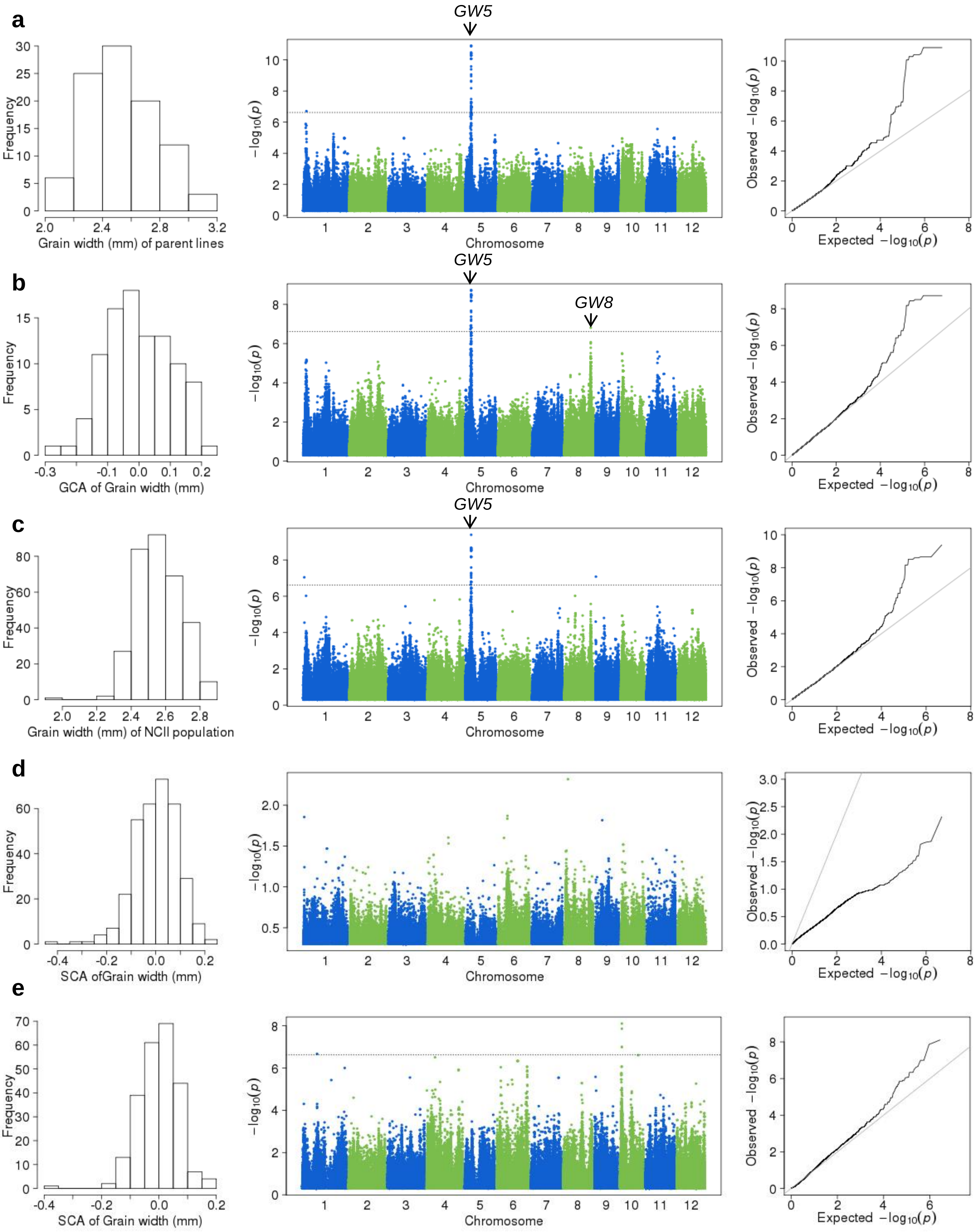

Supplemental Figure 14 Summary of GWAS results for parental grain width (**a**), parental GCA of grain width (**b**), F_1 grain width (**c**), F_1 SCA of grain width (**d, e**). The three frames left to right are phenotypic distribution, Manhattan plots and quantile-quantile (QQ) plots. GWAS in (**a-d**) was based on an additive model and for (**e**) a pseudo non-additive model was used.

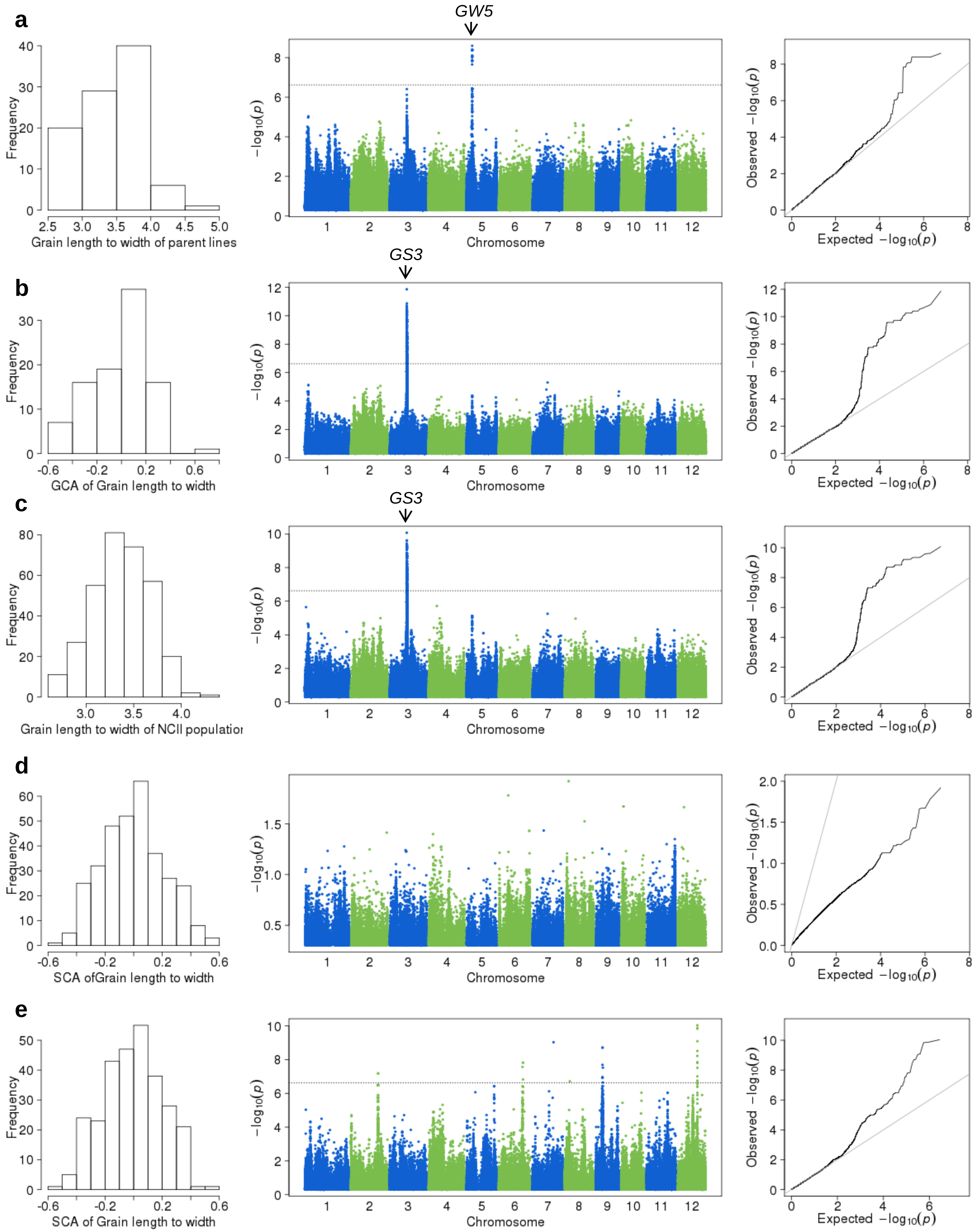

Supplemental Figure 15 Summary of GWAS results for parental grain length to width (**a**), parental GCA of grain length to width (**b**), F_1 grain length to width (**c**), F_1 SCA of grain length to width (**d, e**). The three frames left to right are phenotypic distribution, Manhattan plots and quantile-quantile (QQ) plots. GWAS in (**a-d**) was based on an additive model and for (**e**) a pseudo non-additive model was used.

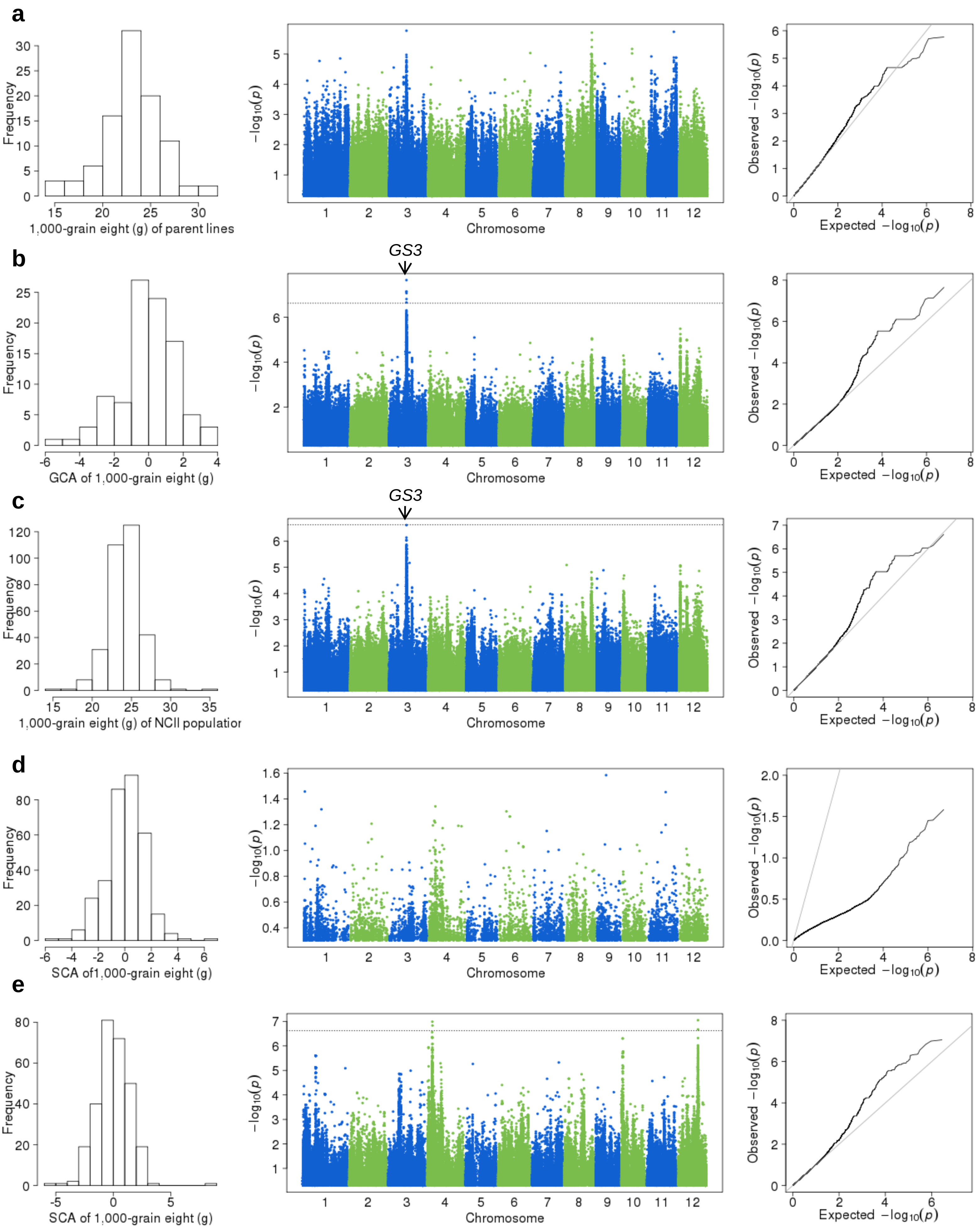

Supplemental Figure 16 Summary of GWAS results for parental 1,000-grain weight (**a**), parental GCA of 1,000-grain weight (**b**), F_1 1,000-grain weight (**c**), F_1 SCA of 1,000-grain weight (**d, e**). The three frames left to right are phenotypic distribution, Manhattan plots and quantile-quantile (QQ) plots. GWAS in (**a-d**) was based on an additive model and for (**e**) a pseudo non-additive model was used.

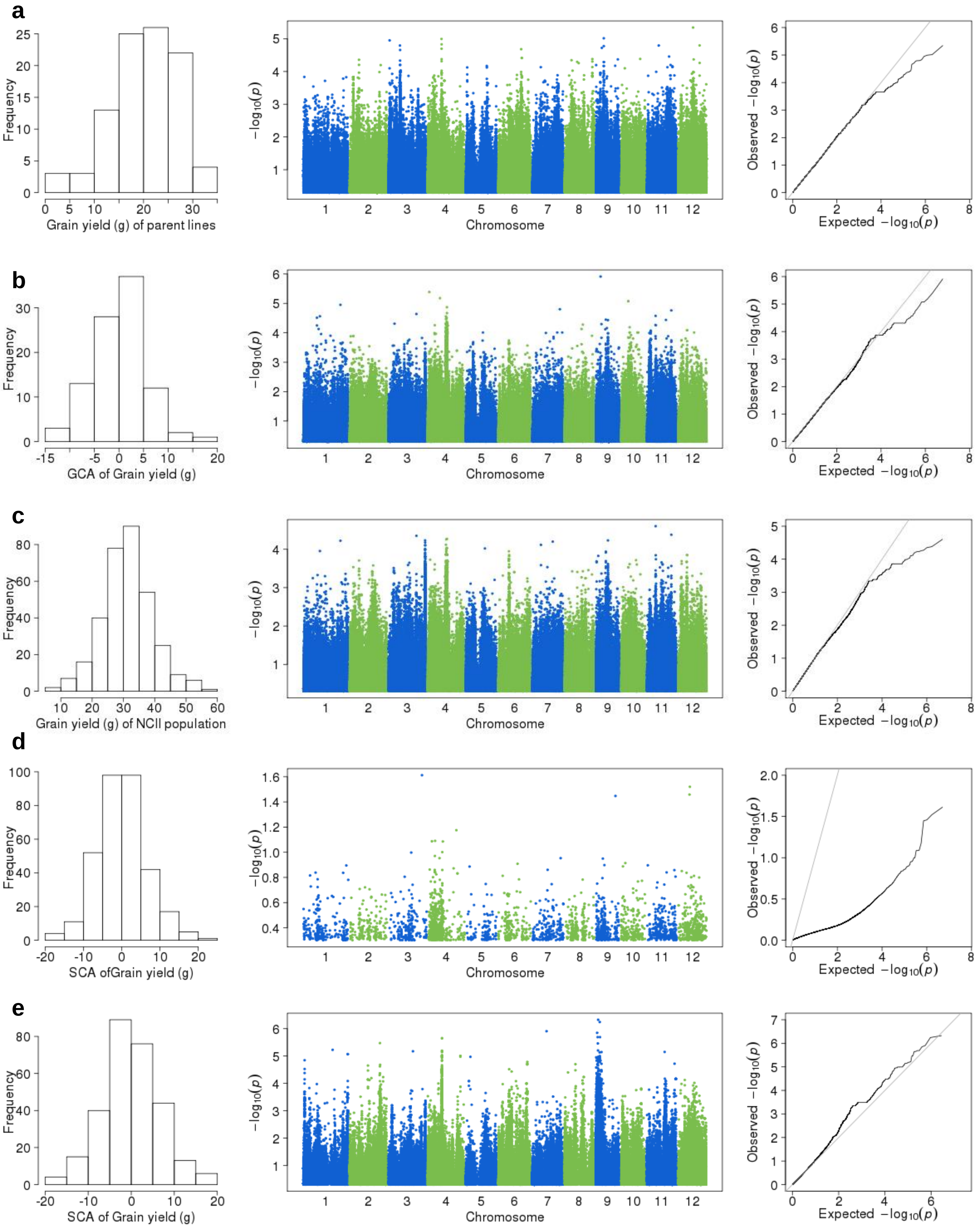

Supplemental Figure 17 Summary of GWAS results for parental grain yield (**a**), parental GCA of grain yield (**b**), F_1 grain yield (**c**), F_1 SCA of grain yield (**d, e**). The three frames left to right are phenotypic distribution, Manhattan plots and quantile-quantile (QQ) plots. GWAS in (**a-d**) was based on an additive model and for (**e**) a pseudo non-additive model was used.

Supplemental Table 1 Estimated effective number of SNPs and significant thresholds in populations

Observed SNPs	Me	Effective ratio	Suggestive *P* value	Significant *P* value
1,663,267	208980.2	0.125644439	4.78514E-06	2.39257E-07

Supplemental Table 2 Significant genome-wide associations for parental yield traits, parental GCA, F_1 yield traits and F_1 SCA of yield traits

parental yield traits

Trait	SNPID	Chr	Pos	Minor/major allele	*P*-value	Known loci	New QTLs
Panicle number	sf0210038291	2	10038291	G/A	2.90E-07		*qPN2*
Flower number	sf0712096308	7	12096308	A/T	1.69E-07		*qPN7*
Grain length	sf0316705162	3	16705162	C/T	2.01E-12	*GS3*	
Grain width	sf0103056595	1	3056595	C/G	1.97E-07		*qGW1*
Grain width	sf0505358848	5	5358848	C/A	1.29E-11	*GW5*	*qGW1*

Parental GCA

Trait	SNPID	Chr	Pos	Minor/major allele	*P*-value	Known loci	New QTLs
Heading date	sf0433335643	4	33335643	A/G	1.07E-07		*qHD4*
Heading date	sf0803953072	8	3953072	A/G	1.12E-11	*Ghd8*	
Heading date	sf1205440147	12	5440147	T/C	1.92E-08		*qHD12*
Panicle length	sf0804017636	8	4017636	C/T	1.56E-08	*Ghd8*	
Panicle number	sf0515997793	5	15997793	C/G	5.07E-08		*qPN5*
Flower number/panicle	sf0316726765	3	16726765	G/A	3.01E-08	*GS3*	
Seed setting rate	sf0417924895	4	17924895	T/A	4.82E-08		*qSSR4*
Grain length	sf0316752029	3	16752029	A/G	1.25E-14	*GS3*	
Grain width	sf0505358848	5	5358848	C/A	1.93E-09	*GW5*	
Grain width	sf0825608959	8	25608959	G/T	1.54E-07	*GW8*	

F_1 yield traits

Trait	SNPID	Chr	Pos	Minor/major allele	*P*-value	Known loci	New QTLs
Heading date	sf0609314861	6	9314861	G/C	5.80E-08	*Hd1*	
Heading date	sf0803953832	8	3953832	A/C	5.61E-09	*Ghd8*	
Seed setting rate	sf0417924895	4	17924895	T/A	8.51E-08		*qSSR4*
Grain length	sf0316752029	3	16752029	A/G	1.39E-12	*GS3*	
Grain width	sf0100945297	1	945297	G/A	9.08E-08		*qGW1*
Grain width	sf0505364539	5	5364539	T/G	4.12E-10	*GW5*	
Grain width	sf0900326946	9	326946	A/T	8.38E-08		*qGW9*

F_1 SCA

Trait	SNPID	Chr	Pos	Minor/major allele	*P*-value	Known loci	New QTLs
Heading date	sf0200711727	2	711727	A/G	9.47E-09		*qHD2*
Heading date	sf0601469352	6	1469352	C/T	2.46E-09	*Hd3a*	
Heading date	sf0609248082	6	9248082	G/T	6.03E-09	*Hd1*	
Flower number/panicle	sf1117317680	11	17317680	T/A	1.14E-07		*qFN11*
Seed setting rate	sf0219043661	2	19043661	G/T	4.45E-08		*qSSR2*
Seed setting rate	sf0903679401	9	3679401	T/C	1.40E-08		*qSSR9*
Panicle length	sf0306338167	3	6338167	G/A	1.27E-07		*qPL3*
Grain length	sf0404634620	4	4634620	T/A	4.43E-08		*qGL4*
Grain length	sf1219265431	12	19265431	T/A	8.52E-13		*qGL12*
Grain width	sf1001788211	10	1788211	G/A	7.87E-09		*qGW10*
1,000-grain weight	sf0404596259	4	4596259	G/A	8.42E-10		*qKWG4*
1,000-grain weight	sf1219264892	12	19264892	G/A	8.94E-08		*qKWG12*

Supplemental Table 3 Agronomic traits of hybrids from cross between four NILs and testers.

	Spikelet number	Grain number	Seed setting	Grain length (mm)	Grain Width (mm)	1,000-grain weight (g)	Panicle number	Panicle length (cm)	Grain yield (g)
ZS97(ghd8)×H1892S	2512±510	1588±436	0.63±0.11	8.11±0.05	2.89±0.03	22.53±0.7	10±2.38	25.8±0.91	35.91±10.5
ZS97(ghd8)×Y58S	2091±396	1390±252	0.66±0.1	8.37±0.11	2.89±0.03	24.02±1.14	12±1.86	30.3±1.42	33.39±6.52
ZS97(ghd8)×GZ63S	2131±530	1180±392	0.54±0.08	8.45±0.11	3.01±0.05	24.51±0.9	9±2.05	26.1±1.32	28.98±9.9
ZS97(ghd8)×HD9802S	2178±600	984±353	0.45±0.05	8.39±0.04	2.88±0.03	24.21±0.35	11±2.86	21.5±0.75	23.88±8.74
ZS97(ghd8)×Chuang5S	2896±599	2001±504	0.68±0.05	8.09±0.09	2.89±0.03	23.37±0.44	13±4.35	23.9±0.93	46.76±11.74
ZS97(ghd8)×B7	2257±673	1458±435	0.65±0.05	7.88±0.07	2.94±0.02	23.82±0.42	11±2.94	23.6±1.3	34.66±10.16
ZS97(ghd8)×YueTaiB	1485±417	874±310	0.58±0.08	8.38±0.33	2.73±0.24	23.3±2.13	7±1.7	23.6±1.84	20.7±7.88
ZS97(ghd8)×HuHan1B	2415±897	1161±491	0.48±0.06	8.89±0.7	3.01±0.07	27.43±1.25	10±4.14	24.5±0.63	31.68±12.96
ZS97(ghd8)×LianxiangB	2384±650	871±323	0.36±0.07	8.76±0.48	2.68±0.27	23.14±2.32	10±2.16	28±1.65	20.29±7.74
ZS97(ghd8)×YixiangB	1842±482	898±585	0.45±0.18	8.42±0.33	2.77±0.16	24.34±1.13	11±2.85	24.8±1.51	21.32±12.9
ZS97(ghd8)×Shen97B	1716±340	964±190	0.57±0.06	8.63±0.04	2.76±0.03	23.69±0.43	10±2.28	26.9±1.09	22.85±4.55
ZS97(ghd8)×GanxiangB	2156±605	1250±308	0.59±0.07	8.38±0.12	2.92±0.11	24.97±1.05	12±3.43	23.4±1.23	31.17±7.65
ZS97(Ghd8)×H1892S	3122±711	1705±363	0.55±0.04	8.2±0.07	2.86±0.03	24.13±0.54	14±4.67	26±0.72	41.18±8.87
ZS97(Ghd8)×Y58S	3103±720	2162±551	0.72±0.1	8.04±0.05	2.91±0.02	23.45±0.42	13±3.16	31±1.14	50.43±12.55
ZS97(Ghd8)×GZ63S	-	-	-	-	-	-	-	-	-
ZS97(Ghd8)×HD9802S	1781±599	1252±504	0.69±0.05	8.36±0.09	2.82±0.03	23.64±0.44	12±4.35	21.3±0.93	29.53±11.74
ZS97(Ghd8)×Chuang5S	3023±1045	2029±690	0.67±0.04	7.93±0.07	2.91±0.03	22.69±0.43	12±4.29	23.1±1.31	46.04±15.84
ZS97(Ghd8)×B7	2500±599	1685±504	0.68±0.05	8.09±0.09	2.98±0.03	24.42±0.44	12±4.35	25.4±0.93	41.18±11.74
ZS97(Ghd8)×YueTaiB	-	-	-	-	-	-	-	-	-
ZS97(Ghd8)×HuHan1B	2715±506	1364±378	0.52±0.24	9.36±0.58	2.64±0.07	22.96±4.34	14±2.12	26.9±4.01	32.13±14.61
ZS97(Ghd8)×LianxiangB	2599±610	1136±358	0.43±0.06	8.73±0.06	2.86±0.03	24.94±0.49	10±2.13	30.7±0.9	26.07±5.64
ZS97(Ghd8)×YixiangB	2278±492	1481±384	0.64±0.08	8.75±0.05	2.75±0.03	23.72±0.98	11±1.73	26.7±1.09	35.27±9.46
ZS97(Ghd8)×Shen97B	2115±841	1210±527	0.56±0.06	8.24±0.26	3.02±0.15	25.14±0.88	13±4.93	28.4±0.51	30.57±13.58

ZS97(Ghd8)×GanxiangB	2138±624	1559±494	0.72±0.07	8.28±0.12	2.89±0.13	24.3±0.36	11±2.22	23.6±1.41	37.54±12.82
MH63(gs3)×1892S	2782±856	1934±700	0.69±0.07	9.04±0.07	2.67±0.04	24.28±0.46	12±3.5	25.9±0.91	47.16±17.73
MH63(gs3)×Y58S	2373±739	1645±478	0.7±0.06	9.02±0.12	2.68±0.04	22.93±0.29	12±3.58	30.1±1.32	37.7±11
MH63(gs3)×GZ63S	2293±528	1603±420	0.7±0.07	9.37±0.07	2.74±0.05	25.23±0.97	11±2.36	27.9±0.8	41.21±11.81
MH63(gs3)×HD9802S	2129±661	1636±544	0.76±0.07	9.39±0.25	2.74±0.08	25.7±1.71	12±3.71	25.2±1.21	42.1±14.06
MH63(gs3)×C815S	2304±679	1371±425	0.6±0.07	9.17±0.09	2.93±0.04	24.95±0.4	12±3.63	26.5±0.82	34.2±10.61
MH63(gs3)×ZS97B	2353±619	1161±589	0.7±0.12	8.47±0.42	3.16±0.14	27.14±1.34	9±3.21	24.2±2.12	31.5±17.14
MH63(gs3)×YueTaiB	2379±399	1888±270	0.8±0.16	9.46±0.06	2.71±0.04	25.7±0.68	11±2.52	24.4±1.7	48.51±6.79
MH63(gs3)×HuHan1B	2126±560	1609±436	0.75±0.04	10.14±0.19	2.95±0.04	30.99±0.29	14±3.08	26.8±1.03	49.89±13.6
MH63(gs3)×LianxiangB	2770±942	2255±641	0.87±0.27	9.5±0.06	2.63±0.15	24.44±2.01	13±3	29.7±1.28	55.77±17.43
MH63(gs3)×FeigaiB	2285±847	1769±736	0.77±0.05	9.2±0.17	2.65±0.14	23.59±1.67	11±4.43	26.5±1.1	41.69±17.74
MH63(GS3)×1892S	2623±367	2060±351	0.78±0.06	8.71±0.07	2.76±0.03	24.09±0.56	14±2.42	27.6±0.99	49.59±8.46
MH63(GS3)×Y58S	3490±866	3032±829	0.86±0.05	8.56±0.08	2.71±0.02	22.73±0.26	17±4.36	31.3±1.08	68.91±18.94
MH63(GS3)×GZ63S	2569±755	2092±606	0.82±0.03	8.97±0.13	2.81±0.04	26.24±0.75	12±3.55	28±0.91	54.76±15.22
MH63(GS3)×HD9802S	2221±708	1573±570	0.73±0.19	8.96±0.09	2.8±0.06	24.6±1.08	13±3.46	23.8±1.6	38.57±13.58
MH63(GS3)×C815S	2270±669	1573±469	0.69±0.04	8.8±0.08	2.9±0.05	24.58±0.56	10±2.87	27±1.1	38.61±11.39
MH63(GS3)×ZS97B	2354±763	1827±783	0.75±0.11	8.15±0.41	3.16±0.03	25.9±2.3	12±3.68	26.2±1.89	47.31±23.13
MH63(GS3)×YueTaiB	2985±664	2410±529	0.81±0.03	8.64±0.15	2.67±0.02	23.65±0.68	13±2.99	30±11.78	56.8±11.78
MH63(GS3)×HuHan1B	3537±706	2762±627	0.78±0.02	8.92±0.35	2.51±0.3	22.69±4.44	14±6.36	28±0.94	62.67±24.98
MH63(GS3)×LianxiangB	2830±972	1991±746	0.7±0.04	9.08±0.13	2.76±0.07	24.62±1.08	12±3.26	29.3±2.17	48.77±17.31
MH63(GS3)×FeigaiB	3012±939	2245±756	0.74±0.07	8.79±0.06	2.4±0.21	21.39±2.24	14±4.11	34±17.65	49.3±21.12

Four NILs were ZS97(*Ghd8*), ZS97(*ghd8*), MH63(*GS3*) and MH63(*gs3*). ZS97 and MH63 respectively indicate the *indica* cultivars Zhenshan97 and Minghui63.

Supplemental Table 4 Phenotype data of parents and F_1 in the study.

Relationship	Indvidual ID	Plant height	Heading date	Spikelet length	Grain length	Grain width	Grain length to width	Effective panicles	Flower number	Flowers per panicle	Seed setting rate	Grain number	1,000 grain weight	Grain yield
Male parent	6	96.33	78.33	23.90	7.16	2.05	3.57	11.30	1744.93	154.42	0.61	1049.43	15.36	16.12
Male parent	7	100.33	78.67	25.18	7.09	2.02	3.60	14.50	2128.43	146.79	0.64	1346.93	15.02	20.26
Male parent	10	96.33	88.00	26.13	8.41	2.31	3.71	5.95	1065.30	179.04	0.30	325.40	20.84	6.77
Male parent	31	100.33	82.67	25.47	9.13	2.53	3.72	8.30	1415.57	170.55	0.73	1034.67	25.06	25.93
Male parent	36	94.33	79.67	19.07	8.98	2.85	3.18	13.50	1159.87	85.92	0.50	588.97	27.68	16.22
Male parent	37	100.00	77.33	22.31	7.41	2.90	2.59	11.27	1515.77	134.54	0.59	860.00	23.02	19.78
Male parent	38	115.33	88.00	28.65	8.67	2.43	3.65	8.70	1462.87	168.15	0.79	1145.40	23.51	26.93
Male parent	41	115.33	79.67	24.06	7.12	2.88	2.50	6.17	1359.17	220.41	0.78	1064.27	21.78	23.41
Male parent	43	110.67	89.00	25.15	7.84	3.02	2.60	8.57	1204.00	140.54	0.80	981.70	27.15	26.76
Male parent	44	100.00	83.67	24.47	9.00	2.88	3.20	8.50	1174.07	138.13	0.55	641.50	28.77	18.47
Male parent	45	106.00	85.67	22.96	6.97	2.12	3.37	9.27	2065.50	222.90	0.85	1760.93	16.29	28.65
Male parent	46	99.67	79.67	27.27	8.98	2.37	3.93	11.07	1677.10	151.55	0.82	1375.50	20.76	28.51
Male parent	49	114.67	86.33	27.49	9.62	2.70	3.75	10.60	1358.00	128.11	0.45	596.97	27.40	16.37
Male parent	50	110.33	93.33	27.03	9.22	2.34	4.02	9.30	1613.70	173.52	0.67	1087.40	23.70	25.64
Male parent	53	120.33	91.33	27.94	9.17	2.53	3.73	7.73	1058.97	136.94	0.45	467.67	24.88	11.84
Male parent	60	115.33	92.67	25.66	7.82	2.47	3.21	9.47	1435.27	151.61	0.52	735.10	20.86	15.35
Male parent	61	115.33	89.00	30.70	8.68	2.47	3.58	8.85	1585.57	179.16	0.61	957.07	26.35	25.24
Male parent	118	104.33	102.00	24.07	8.17	2.33	3.58	8.13	1440.87	177.16	0.37	540.50	21.27	11.52
Male parent	138	105.67	89.00	26.58	8.67	2.66	3.22	7.13	1163.00	163.19	0.47	580.05	25.55	13.82
Male parent	147	114.67	93.00	26.63	9.26	2.64	3.58	10.10	1516.67	150.17	0.58	873.33	27.69	24.20
Male parent	148	116.00	88.67	24.21	8.98	2.52	3.69	6.67	1161.40	174.21	0.65	780.97	27.76	21.93
Male parent	151	99.00	86.67	23.47	7.64	2.75	2.81	9.77	1915.13	196.09	0.72	1398.30	22.64	31.50
Male parent	164	99.00	71.00	26.77	7.67	2.97	2.63	6.17	1460.33	236.81	0.58	877.17	24.49	21.57
Male parent	165	110.33	73.00	23.76	7.38	2.92	2.57	8.57	1408.50	164.42	0.59	837.73	23.76	19.85
Male parent	166	105.67	82.33	24.05	8.07	2.74	3.00	7.17	1673.27	233.48	0.70	1191.00	24.41	28.69
Male parent	169	120.00	95.67	28.65	7.53	2.69	2.85	7.83	1416.33	180.81	0.87	1233.10	22.06	27.19
Male parent	170	95.67	76.33	23.24	7.12	2.89	2.52	12.83	1396.43	108.81	0.67	928.77	22.43	20.82
Male parent	173	114.00	78.33	23.63	7.57	2.80	2.74	8.40	1195.83	142.36	0.52	599.33	22.66	13.42
Male parent	175	111.00	92.33	26.62	8.78	2.29	3.95	7.80	1604.60	205.72	0.60	974.27	22.62	22.04
Male parent	178	107.00	89.33	25.95	7.50	2.69	2.81	6.83	1295.53	189.59	0.66	853.93	22.99	19.75
Male parent	W017	114.67	90.67	24.92	9.16	3.08	3.00	6.00	752.50	125.42	0.71	531.00	30.23	16.05
Male parent	W040	120.33	95.00	27.92	8.48	2.45	3.55	6.60	1192.00	180.61	0.16	183.30	18.49	3.45

Male parent	W044	99.00	98.00	24.41	9.09	2.62	3.55	8.90	1149.17	129.12	0.59	679.80	28.91	19.68
Male parent	W046	95.33	102.33	23.85	8.38	2.97	2.83	11.07	1222.70	110.48	0.64	793.13	24.55	19.47
Male parent	W049	154.67	86.33	29.36	7.45	2.12	3.62	9.73	2004.57	205.95	0.83	1662.07	16.42	27.31
Male parent	W066	94.33	81.33	23.82	8.03	2.37	3.46	13.13	1301.27	99.08	0.19	252.50	17.33	4.33
Male parent	W078	98.67	103.33	23.25	7.74	2.56	3.10	8.00	1133.60	141.70	0.68	775.20	23.41	18.11
Male parent	W083	114.33	85.33	25.83	8.60	2.62	3.34	9.00	1346.60	149.62	0.76	1029.17	26.12	26.91
Male parent	W086	109.33	85.33	25.92	8.77	2.42	3.72	9.43	1642.17	174.08	0.73	1195.33	23.45	27.95
Male parent	W118	81.00	86.33	21.23	9.48	2.24	4.29	5.55	870.27	156.80	0.34	293.57	25.81	7.60
Male parent	W137	109.00	100.33	26.10	9.16	3.17	2.93	10.65	1466.10	137.66	0.65	955.40	30.06	28.66
Male parent	W138	99.00	72.67	19.93	7.65	2.41	3.24	14.67	1528.67	104.23	0.59	884.50	23.97	20.36
Male parent	W147	115.33	86.67	24.85	8.35	2.50	3.39	8.35	1250.10	149.71	0.55	687.47	22.98	15.80
Male parent	W149	105.33	79.00	23.74	9.04	2.77	3.31	11.50	1307.03	113.66	0.40	533.07	25.44	13.92
Male parent	W153	99.67	92.67	25.43	8.94	2.52	3.63	8.43	1190.57	141.17	0.65	769.07	25.10	19.32
Male parent	W156	113.33	81.33	27.15	7.85	2.90	2.78	9.60	2021.40	210.56	0.76	1529.80	23.00	34.30
Male parent	W159	99.67	80.33	23.84	7.88	2.63	3.06	8.57	1398.67	163.27	0.83	1156.97	21.79	25.27
Male parent	W161	91.00	75.67	23.69	8.06	2.57	3.17	12.17	1908.67	156.88	0.71	1432.00	22.94	33.20
Male parent	W168	106.33	96.33	27.32	8.40	2.68	3.22	8.23	1441.40	175.07	0.52	772.30	25.52	19.82
Male parent	W169	99.67	89.33	25.78	8.26	2.45	3.43	10.50	1689.57	160.91	0.47	796.57	22.32	17.83
Male parent	W170	100.33	87.00	25.58	8.61	2.48	3.57	9.80	1687.67	172.21	0.67	1137.60	24.19	27.53
Male parent	W172	109.00	85.67	28.27	8.74	2.27	3.95	9.23	1628.93	176.42	0.65	1097.97	23.82	26.14
Male parent	W176	97.67	89.00	25.09	7.81	2.70	2.91	10.17	1525.70	150.07	0.65	998.10	23.58	23.72
Male parent	W177	99.33	87.33	26.17	7.83	2.76	2.89	8.65	1504.30	173.91	0.63	954.87	23.15	22.10
Male parent	W178	104.33	105.33	24.37	7.92	2.40	3.37	10.03	1813.93	180.79	0.78	1427.00	19.59	28.44
Male parent	W181	105.33	90.67	29.06	8.46	2.51	3.50	14.13	1862.53	131.78	0.41	804.40	22.70	18.61
Male parent	W183	104.33	92.33	25.47	8.39	2.25	3.81	11.63	1176.40	101.12	0.49	574.77	21.64	11.99
Male parent	W184	97.33	79.67	24.70	8.19	2.47	3.39	13.50	1514.17	112.16	0.55	791.93	22.23	17.56
Male parent	W185	100.67	100.33	25.24	8.80	2.20	4.11	9.73	1319.87	135.60	0.57	747.33	22.11	16.45
Male parent	W190	107.00	98.33	29.68	8.58	2.58	3.39	7.85	1294.13	164.86	0.30	396.63	27.22	10.88
Male parent	W191	122.33	114.00	31.85	9.51	2.00	4.88	10.60	1526.80	144.04	0.63	987.80	21.20	21.07
Male parent	W194	99.67	76.00	22.27	8.61	2.26	3.88	9.73	1309.10	134.50	0.59	779.77	19.70	15.31
Male parent	W199	117.67	98.00	32.86	9.73	2.71	3.68	8.00	1321.07	165.13	0.57	754.70	27.96	21.11
Male parent	W200	108.00	81.33	25.73	9.14	2.48	3.78	8.10	1354.77	167.26	0.43	632.20	23.01	15.58
Male parent	W221	122.00	106.00	22.82	8.38	2.49	3.45	8.80	1380.77	156.91	0.72	1004.80	20.79	21.14
Male parent	W222	115.00	90.67	29.58	8.97	2.39	3.85	8.85	1460.00	164.97	0.30	433.63	24.67	10.69
Male parent	W223	110.00	85.33	25.95	8.61	2.81	3.11	9.13	1719.13	188.23	0.38	787.80	26.36	20.90

Male parent	W224	111.33	76.67	26.26	7.76	2.25	3.51	10.27	1984.00	193.25	0.80	1592.40	19.34	30.73
Male parent	W225	87.33	90.00	24.08	8.76	2.28	3.90	5.20	765.60	147.23	0.36	271.80	23.02	6.29
Male parent	W227	110.00	81.00	26.68	9.34	2.38	4.05	9.93	1349.27	135.83	0.81	1094.60	26.08	28.60
Male parent	W229	123.33	85.33	27.35	9.19	2.40	3.96	9.47	1379.57	145.73	0.74	1028.40	24.10	24.71
Male parent	W230	112.00	98.67	26.36	8.48	2.60	3.34	9.70	1566.17	161.46	0.70	1096.30	20.88	23.02
Male parent	W231	116.67	90.67	28.08	8.02	2.57	3.15	7.23	1183.00	163.55	0.74	886.03	22.90	20.26
Male parent	W232	107.33	87.00	23.87	7.79	2.54	3.10	9.05	1598.47	176.63	0.71	1132.00	22.02	24.97
Male parent	W233	125.00	78.00	23.64	7.37	2.68	2.79	5.87	1054.03	179.66	0.53	557.10	21.99	12.30
Male parent	W236	97.67	86.00	27.71	9.06	2.59	3.56	8.87	1738.17	196.03	0.49	883.43	25.79	23.02
Male parent	W237	117.67	96.00	28.52	8.88	2.29	4.00	9.65	1689.67	175.09	0.34	580.70	22.98	13.37
Male parent	W240	119.67	79.67	25.37	8.49	2.64	3.26	10.30	1276.60	123.94	0.39	489.83	24.64	11.94
Male parent	W242	120.00	103.33	23.57	8.53	2.44	3.56	8.80	1384.80	157.36	0.52	718.60	24.79	17.81
Male parent	W263	119.67	98.33	28.17	7.91	2.65	3.03	7.00	1644.87	234.98	0.77	1262.17	20.04	25.24
Male parent	W265	127.67	96.33	28.07	8.11	2.70	3.03	9.80	1996.77	203.75	0.70	1393.50	20.43	28.47
Male parent	W267	129.00	69.00	22.34	8.56	2.60	3.40	8.17	891.40	109.15	0.61	538.97	24.95	13.39
Male parent	W269	90.00	73.67	24.22	8.72	2.27	3.90	10.30	1540.00	149.51	0.81	1243.70	19.44	24.32
Male parent	W270	94.67	76.67	19.05	7.22	2.67	2.76	7.87	1276.63	162.28	0.76	964.40	20.76	20.03
Male parent	W293	100.33	101.33	23.54	8.21	2.82	2.94	7.00	1169.60	167.09	0.78	910.60	25.36	23.04
Male parent	W295	102.00	90.67	26.04	8.68	2.26	3.94	11.77	1200.73	102.05	0.49	580.87	22.40	13.04
Male parent	W298	95.33	96.67	24.09	8.23	2.34	3.56	11.90	1528.07	128.41	0.76	1162.93	20.50	23.83
Male parent	W301	108.33	91.67	26.86	9.72	2.32	4.33	13.37	1295.83	96.95	0.45	586.63	25.19	14.79
Male parent	W304	93.33	93.00	25.22	8.42	2.45	3.64	5.80	1040.80	179.45	0.16	164.40	14.63	2.44
Male parent	W308	104.67	102.67	26.54	8.75	2.39	3.72	10.25	1226.37	119.65	0.79	969.47	23.63	22.90
Male parent	W309	110.67	103.67	26.56	9.13	2.37	4.00	11.00	1246.40	113.31	0.73	912.60	24.32	22.22
Male parent	W310	109.67	84.67	28.10	8.02	2.27	3.60	10.90	1657.07	152.02	0.59	970.77	19.31	18.74
Male parent	W311	112.00	103.00	25.70	8.49	2.48	3.50	9.03	1802.43	199.53	0.73	1312.10	21.15	27.67
Male parent	W312	117.00	86.00	26.86	8.51	2.34	3.73	9.40	1407.27	149.71	0.68	961.83	22.49	21.82
Male parent	W315	104.33	90.00	25.59	8.66	2.33	3.81	9.37	1177.53	125.72	0.70	850.20	23.67	19.74
Male parent	W317	110.33	94.67	27.47	8.77	2.49	3.61	8.85	1568.80	177.27	0.69	1081.80	23.54	25.64
Male parent	r287	111.00	65.00	26.16	9.02	2.25	4.13	7.83	1131.83	144.49	0.67	756.83	20.10	15.25
Female paren	H815S	104.67	87.67	NA	NA	NA	NA	NA	NA	NA	NA	NA	NA	NA
Female paren	H6S	106.67	82.33	NA	NA	NA	NA	NA	NA	NA	NA	NA	NA	NA
Female paren	H2613S	103.33	84.67	NA	NA	NA	NA	NA	NA	NA	NA	NA	NA	NA
Female paren	H9802S	101.67	80.67	NA	NA	NA	NA	NA	NA	NA	NA	NA	NA	NA
F_1	6-2613s	104.67	78.67	24.96	8.10	2.43	3.39	13.83	1915.77	140.08	0.55	1054.10	21.08	22.23

F_1	7-2613s	101.33	79.33	25.51	7.88	2.35	3.40	11.67	2150.43	180.92	0.71	1550.43	20.74	32.27
F_1	10-2613s	104.67	80.67	27.66	9.14	2.46	3.80	9.35	2338.50	254.67	0.67	1602.13	25.83	41.75
F_1	31-2613s	101.33	80.33	26.30	9.08	2.57	3.63	9.17	1620.53	179.20	0.70	1117.00	26.17	28.76
F_1	36-2613s	110.67	90.67	22.76	9.32	2.67	3.57	10.67	1458.83	135.36	0.59	862.73	29.27	25.46
F_1	37-2613s	111.00	95.00	26.04	7.96	2.71	3.00	10.33	2347.47	227.49	0.65	1534.13	24.37	37.20
F_1	41-2613s	112.67	76.00	25.57	8.07	2.75	3.01	10.07	1969.33	198.20	0.85	1695.43	24.27	40.99
F_1	43-2613s	107.33	79.67	23.68	8.48	2.72	3.19	11.97	1577.57	135.76	0.45	717.87	22.76	18.48
F_1	44-2613s	108.33	82.33	23.69	9.10	2.61	3.53	7.50	1472.83	195.27	0.86	1278.67	26.99	34.50
F_1	45-2613s	108.00	81.67	23.79	7.75	2.36	3.38	8.67	1914.83	220.88	0.82	1566.77	19.76	30.97
F_1	46-2613s	105.33	79.67	28.53	8.86	2.44	3.73	8.67	1797.07	207.68	0.82	1481.60	23.80	35.26
F_1	49-2613s	130.33	79.67	28.61	8.97	2.65	3.46	9.47	1550.37	163.37	0.87	1349.10	27.04	36.47
F_1	50-2613s	112.33	81.67	26.90	9.33	2.57	3.73	8.80	1771.80	201.34	0.81	1449.30	25.21	36.24
F_1	53-2613s	127.00	77.33	28.65	9.10	2.56	3.62	10.83	2382.43	218.91	0.69	1639.27	27.26	44.45
F_1	60-2613s	106.73	80.00	26.19	8.30	2.56	3.30	9.93	1851.83	186.50	0.62	1175.26	23.14	27.18
F_1	61-2613s	117.67	82.00	29.14	8.89	2.62	3.46	8.30	1598.67	193.23	0.74	1176.47	25.91	30.38
F_1	118-2613s	116.00	90.33	25.92	8.81	2.55	3.55	9.17	1763.97	191.78	0.78	1407.20	24.84	35.14
F_1	147-2613s	115.33	86.00	26.03	9.21	2.58	3.65	10.55	1884.20	178.62	0.82	1542.60	26.80	41.19
F_1	148-2613s	122.33	87.33	27.18	9.02	2.55	3.63	8.50	1943.23	220.13	0.77	1525.37	27.32	41.48
F_1	151-2613s	107.00	78.00	24.95	8.25	2.66	3.15	11.00	2019.77	183.35	0.78	1594.00	24.62	39.26
F_1	164-2613s	99.33	78.67	26.11	8.51	2.79	3.11	8.50	2129.90	248.65	0.73	1558.23	25.85	40.49
F_1	165-2613s	108.00	86.67	26.86	7.94	2.68	3.00	10.25	1660.90	168.79	0.69	1155.50	23.97	27.80
F_1	166-2613s	113.67	78.00	24.41	8.52	2.55	3.43	11.00	1994.70	182.60	0.80	1589.80	25.40	40.30
F_1	169-2613s	120.33	78.33	26.23	8.12	2.68	3.10	9.90	1797.73	182.13	0.89	1599.93	24.75	39.66
F_1	170-2613s	126.33	81.00	24.97	8.04	2.65	3.09	10.97	1869.40	170.65	0.81	1518.87	25.06	38.06
F_1	173-2613s	113.33	79.67	23.75	8.17	2.66	3.09	13.17	2155.93	166.23	0.66	1446.43	25.03	36.06
F_1	175-2613s	126.00	85.67	27.09	9.26	2.46	3.84	11.00	2380.70	215.02	0.59	1451.60	34.12	39.24
F_1	178-2613s	122.00	89.33	26.50	8.42	2.52	3.43	9.27	1890.27	206.66	0.67	1308.60	25.07	32.92
F_1	W017-2613s	125.33	82.33	26.78	9.20	2.90	3.20	10.40	1875.20	180.31	0.28	514.80	28.63	14.74
F_1	W040-2613s	124.00	94.33	27.37	8.95	2.46	3.75	8.17	1663.77	207.52	0.65	1080.43	25.95	27.97
F_1	W044-2613s	103.67	82.00	25.57	9.13	2.60	3.58	8.10	1239.00	152.84	0.72	896.87	28.39	25.49

F_1	W046-2613s	97.67	81.33	25.94	8.48	2.64	3.26	10.00	1821.43	181.46	0.77	1398.27	25.08	35.17
F_1	W049-2613s	162.00	79.67	27.65	8.14	2.34	3.52	11.25	2089.70	186.13	0.79	1659.40	21.30	35.35
F_1	W066-2613s	134.67	78.00	25.99	8.40	2.59	3.31	7.30	1323.40	184.47	0.55	731.60	23.92	17.62
F_1	W078-2613s	114.67	94.33	28.14	8.57	2.53	3.45	9.55	1831.30	193.65	0.82	1510.40	24.74	37.41
F_1	W083-2613s	121.00	85.67	27.39	8.90	2.67	3.43	10.30	1859.30	180.51	0.65	1212.70	27.61	33.53
F_1	W086-2613s	110.67	79.33	25.83	8.92	2.51	3.63	9.25	1592.13	172.18	0.74	1196.00	25.33	30.35
F_1	W118-2613s	113.00	76.33	26.79	9.26	2.47	3.82	8.90	1936.67	215.30	0.39	843.67	25.79	22.15
F_1	W137-2613s	131.33	100.00	28.46	9.15	2.75	3.39	11.10	2141.90	193.90	0.75	1612.07	28.71	46.11
F_1	W138-2613s	103.67	76.33	22.72	8.10	2.48	3.35	11.87	1773.50	149.60	0.69	1247.77	22.73	28.35
F_1	W147-2613s	107.67	78.00	24.71	8.85	2.56	3.52	8.90	1516.20	170.27	0.83	1270.80	26.37	33.39
F_1	W149-2613s	115.33	80.33	24.06	9.25	2.68	3.55	11.43	1609.00	143.80	0.68	1095.17	27.05	29.65
F_1	W153-2613s	110.33	82.67	26.36	9.07	2.61	3.55	9.70	1716.73	178.54	0.78	1345.10	25.95	34.83
F_1	W156-2613s	112.00	81.00	26.25	8.46	2.73	3.16	11.20	2208.30	197.07	0.73	1597.20	26.77	42.66
F_1	W159-2613s	112.67	79.67	26.33	8.51	2.62	3.32	9.33	1793.47	193.20	0.83	1514.33	25.04	38.12
F_1	W161-2613s	113.67	73.33	24.62	8.39	2.51	3.41	11.85	2233.10	188.44	0.84	1879.87	23.82	44.56
F_1	W168-2613s	130.33	96.00	28.96	8.78	2.72	3.38	9.00	1953.90	212.40	0.77	1508.40	23.40	33.80
F_1	W169-2613s	101.33	79.33	24.38	8.66	2.45	3.60	11.00	1808.50	165.64	0.77	1392.50	25.01	34.81
F_1	W170-2613s	105.33	79.33	26.08	8.72	2.56	3.47	9.27	1683.77	181.46	0.73	1242.93	25.39	31.85
F_1	W172-2613s	110.33	77.33	26.04	9.11	2.43	3.85	9.00	1458.50	162.06	0.77	1117.40	27.11	30.25
F_1	W176-2613s	114.00	88.67	26.30	8.02	2.71	3.03	9.50	2038.87	214.28	0.72	1467.97	22.92	33.98
F_1	W177-2613s	107.33	76.67	26.27	7.97	2.68	3.02	8.90	2050.67	233.24	0.64	1331.57	23.06	31.06
F_1	W178-2613s	121.67	96.33	25.35	7.96	2.49	3.28	9.40	1769.67	190.24	0.90	1597.27	21.83	34.28
F_1	W181-2613s	119.67	90.00	27.93	8.80	2.46	3.65	10.13	1784.57	175.63	0.75	1353.20	25.94	35.09
F_1	W183-2613s	111.00	86.67	28.25	8.76	2.44	3.68	10.60	1962.47	185.47	0.68	1327.87	24.93	33.06
F_1	W184-2613s	104.67	74.00	25.22	8.67	2.49	3.55	10.67	1541.80	144.04	0.70	1077.30	25.35	27.28
F_1	W185-2613s	89.67	79.33	25.70	8.83	2.48	3.64	11.20	1905.77	175.70	0.63	1194.77	25.12	30.12
F_1	W190-2613s	128.33	92.00	25.82	9.16	2.50	3.75	9.50	2183.50	229.84	0.74	1626.00	26.78	43.55
F_1	W191-2613s	114.00	80.00	27.76	9.51	2.31	4.23	14.30	2392.80	167.33	0.63	1508.30	24.35	36.73
F_1	W194-2613s	111.00	77.33	24.26	8.96	2.46	3.75	10.13	1959.20	193.89	0.79	1545.23	23.80	36.76
F_1	W199-2613s	118.00	88.33	28.88	7.56	2.78	4.01	8.00	1755.30	219.40	0.66	1158.50	27.55	NA

F_1	W200-2613s	116.67	88.00	27.43	9.07	2.44	3.80	10.53	1651.33	151.84	0.64	1096.00	23.88	26.39
F_1	W221-2613s	116.00	92.33	25.40	8.82	2.53	3.56	9.80	1852.20	189.00	0.77	1420.40	23.77	33.77
F_1	W222-2613s	124.67	84.67	28.57	9.11	2.43	3.82	10.75	1634.17	154.74	0.49	801.40	26.78	21.57
F_1	W223-2613s	114.67	73.33	25.18	8.72	2.77	3.20	10.00	1820.00	182.00	0.39	710.00	27.51	19.53
F_1	W225-2613s	123.67	76.33	25.49	9.22	2.54	3.70	13.80	1408.70	102.08	0.45	647.00	27.11	17.53
F_1	W227-2613s	112.00	78.33	25.00	9.29	2.42	3.93	13.67	1818.30	140.18	0.74	1333.60	27.10	36.59
F_1	W229-2613s	119.00	87.00	30.03	9.06	2.45	3.80	11.17	2389.83	212.33	0.69	1629.00	25.04	41.06
F_1	W230-2613s	123.67	90.67	27.74	8.65	2.50	3.56	9.83	1941.10	197.24	0.74	1439.47	24.20	34.97
F_1	W231-2613s	124.00	76.33	26.11	8.46	2.48	3.50	8.40	1450.40	172.98	0.78	1135.90	24.75	28.11
F_1	W232-2613s	112.00	80.67	25.29	7.90	2.47	3.26	9.20	1919.80	208.67	0.71	1359.20	22.51	30.50
F_1	W233-2613s	121.00	78.00	24.12	8.15	2.71	3.04	10.20	1977.00	193.82	0.82	1610.60	25.08	40.36
F_1	W236-2613s	118.33	79.00	28.05	9.09	2.57	3.62	7.30	1652.50	224.14	0.81	1349.87	26.78	36.12
F_1	W237-2613s	106.33	89.33	29.07	8.86	2.45	3.73	9.03	1915.87	212.49	0.82	1570.83	23.96	37.70
F_1	W240-2613s	113.00	78.33	24.56	9.19	2.58	3.63	14.53	1784.20	114.91	0.67	1087.67	28.81	30.96
F_1	W242-2613s	123.00	76.33	24.89	8.34	2.72	3.12	10.25	1733.33	168.34	0.65	1176.50	25.57	30.06
F_1	W263-2613s	123.00	90.00	27.29	8.29	2.67	3.17	8.67	1883.17	217.62	0.93	1760.00	23.74	41.82
F_1	W267-2613s	115.33	68.33	25.27	8.94	2.56	3.57	10.95	1500.57	137.58	0.79	1201.30	26.30	31.47
F_1	W269-2613s	113.33	72.00	25.48	8.91	2.46	3.70	12.40	2490.20	200.82	0.85	2122.40	23.76	50.57
F_1	W270-2613s	116.17	73.67	22.81	7.79	2.63	3.02	12.53	2430.66	194.00	0.78	1870.56	22.99	41.83
F_1	W293-2613s	102.33	81.33	24.96	8.61	2.69	3.30	9.50	1679.33	176.66	0.78	1311.53	25.89	33.77
F_1	W295-2613s	112.00	86.00	27.66	8.95	2.43	3.81	8.55	1369.87	160.52	0.74	1008.97	25.91	26.15
F_1	W298-2613s	113.00	73.33	25.65	8.80	2.48	3.65	11.93	2132.77	176.84	0.76	1617.77	24.34	39.26
F_1	W301-2613s	117.33	87.00	27.28	9.44	2.41	4.03	11.00	1321.33	120.12	0.55	745.33	26.84	20.11
F_1	W304-2613s	116.67	81.33	26.34	8.24	2.64	3.19	9.80	2564.98	261.84	0.57	1499.79	22.59	33.15
F_1	W308-2613s	122.33	92.33	28.35	9.08	2.53	3.70	9.83	1658.73	170.26	0.79	1307.23	25.80	33.73
F_1	W309-2613s	125.67	84.67	28.48	9.16	2.45	3.83	11.63	1938.27	167.12	0.84	1618.50	25.75	41.68
F_1	W310-2613s	126.00	88.00	28.89	8.65	2.44	3.64	9.90	1931.57	193.70	0.78	1519.13	23.70	36.05
F_1	W311-2613s	107.00	82.00	25.61	8.66	2.45	3.58	11.17	2299.67	205.87	0.81	1849.83	24.47	45.28
F_1	W312-2613s	128.00	86.67	28.99	8.82	2.43	3.67	8.50	1630.00	195.63	0.70	1144.00	25.68	29.29
F_1	W315-2613s	115.67	89.00	27.34	8.95	2.47	3.69	9.20	1738.43	188.66	0.65	1154.93	26.35	30.39

F_1	W317-2613s	120.67	86.67	27.30	8.97	2.52	3.63	11.05	2044.10	185.06	0.64	1311.17	26.39	34.58
F_1	R287-2613s	114.33	70.67	26.35	8.71	2.39	3.75	8.83	1887.03	212.64	0.80	1512.47	23.50	35.16
F_1	6-6s	97.00	78.00	26.62	7.49	2.43	3.13	13.50	1691.80	129.98	0.64	1084.03	19.04	20.61
F_1	7-6s	90.00	77.00	26.80	7.48	2.41	3.16	14.73	2159.50	148.62	0.70	1549.23	19.37	30.00
F_1	10-6s	97.00	79.33	29.85	8.42	2.58	3.33	8.17	1802.10	221.02	0.58	1060.27	23.05	24.48
F_1	31-6s	90.00	80.67	27.02	8.62	2.65	3.32	8.43	1356.33	161.18	0.83	1130.00	24.79	28.10
F_1	36-6s	101.33	87.67	23.88	7.89	2.72	2.97	8.10	1076.37	134.18	0.65	742.17	23.64	15.96
F_1	37-6s	104.33	83.67	27.55	7.42	2.73	2.75	9.37	1693.33	179.86	0.81	1384.87	22.58	31.31
F_1	38-6s	112.67	140.00	NA	NA	NA	NA	NA	NA	NA	NA	NA	NA	NA
F_1	41-6s	99.67	79.33	26.83	7.70	2.84	2.73	8.33	1678.60	200.18	0.78	1325.70	23.21	30.68
F_1	43-6s	104.67	79.33	26.39	7.68	2.83	2.73	9.53	1520.40	159.79	0.86	1305.90	24.15	31.64
F_1	44-6s	112.00	81.67	27.96	8.12	2.69	3.08	9.25	1684.50	181.93	0.80	1356.87	23.83	32.49
F_1	45-6s	104.33	80.33	26.14	7.36	2.47	3.03	11.50	2121.00	184.22	0.89	1881.07	19.32	36.34
F_1	46-6s	97.67	78.67	28.59	8.41	2.60	3.31	10.97	1727.73	157.34	0.77	1319.03	23.53	31.07
F_1	49-6s	121.33	91.00	29.73	8.72	2.68	3.30	7.70	1314.97	171.62	0.69	907.30	25.38	23.19
F_1	50-6s	97.67	84.67	29.40	8.81	2.58	3.50	10.00	1754.00	175.40	0.68	1192.00	23.64	28.20
F_1	53-6s	106.33	88.67	28.95	8.83	2.69	3.37	8.50	1526.30	180.47	0.69	1057.63	26.54	28.11
F_1	60-6s	111.00	84.67	28.07	7.65	2.58	2.99	6.70	1532.47	231.86	0.64	983.13	20.69	20.37
F_1	61-6s	107.33	83.33	31.73	8.36	2.61	3.25	10.90	1968.07	180.56	0.78	1538.17	24.45	37.65
F_1	118-6s	109.67	90.67	28.21	8.36	2.61	3.27	7.80	1563.57	198.52	0.56	913.40	23.00	21.41
F_1	147-6s	104.00	87.00	29.03	8.75	2.69	3.32	10.20	1692.50	165.87	0.61	1034.40	25.99	26.91
F_1	148-6s	110.33	93.00	28.50	8.43	2.62	3.28	8.30	1578.83	189.70	0.80	1268.50	24.94	31.87
F_1	151-6s	99.67	79.33	25.65	7.77	2.78	2.80	10.50	1816.00	172.57	0.77	1410.30	22.68	32.02
F_1	165-6s	102.00	84.00	27.89	7.59	2.80	2.74	8.50	1690.90	198.38	0.77	1284.60	23.18	29.72
F_1	166-6s	95.00	79.00	28.56	8.51	2.77	3.18	9.85	1780.83	182.56	0.75	1350.50	25.04	33.94
F_1	169-6s	102.33	78.33	29.12	7.64	2.71	2.85	9.20	1606.57	174.97	0.85	1367.13	23.39	31.95
F_1	170-6s	101.00	79.33	25.45	7.34	2.74	2.71	11.87	1582.23	132.41	0.84	1326.83	21.98	29.25
F_1	175-6s	108.00	89.00	28.28	8.40	2.46	3.48	8.35	1515.07	181.70	0.80	1219.27	23.08	28.12
F_1	178-6s	100.00	89.33	27.47	7.63	2.80	2.79	7.27	1201.37	167.71	0.80	958.27	22.17	21.29
F_1	W017-6s	115.33	82.67	28.53	8.36	2.87	2.97	10.75	1999.30	186.47	0.48	957.67	25.94	24.81

F_1	W044-6s	107.33	96.67	29.36	8.48	2.49	3.50	10.50	2218.00	211.24	0.69	1522.50	23.11	35.18
F_1	W046-6s	119.00	95.67	28.02	8.20	2.77	3.00	9.93	1825.67	185.61	0.75	1360.33	23.19	31.62
F_1	W049-6s	160.00	80.33	30.06	7.89	2.38	3.37	8.43	1876.60	222.66	0.87	1632.37	20.37	33.18
F_1	W078-6s	106.67	94.33	27.73	8.12	2.66	3.09	10.50	1779.30	170.52	0.73	1293.70	22.99	29.77
F_1	W083-6s	110.00	84.33	29.43	8.62	2.73	3.25	9.83	1725.66	175.50	0.67	1163.45	26.74	31.44
F_1	W086-6s	107.67	80.00	26.19	8.85	2.58	3.50	10.00	1744.50	174.45	0.72	1255.80	23.77	29.88
F_1	W118-6s	98.00	76.67	26.99	9.00	2.68	3.44	7.60	1235.10	167.33	0.40	491.17	25.12	12.53
F_1	W137-6s	123.33	103.67	30.26	8.46	2.84	3.03	9.60	2217.30	230.97	0.85	1885.30	26.91	50.69
F_1	W138-6s	101.00	79.67	25.80	7.63	2.58	3.01	10.73	1787.37	166.73	0.75	1344.13	21.55	28.87
F_1	W147-6s	105.67	79.00	27.26	8.25	2.64	3.19	9.55	1731.00	181.03	0.82	1417.30	24.22	34.30
F_1	W149-6s	121.33	80.00	27.32	8.92	2.59	3.60	12.90	2364.90	181.73	0.73	1747.40	25.33	44.44
F_1	W153-6s	116.00	87.00	28.39	8.60	2.67	3.29	12.67	1995.37	158.75	0.82	1669.83	25.17	42.41
F_1	W156-6s	105.00	81.67	27.58	7.86	2.76	2.90	8.85	1497.00	168.65	0.79	1173.50	24.91	29.25
F_1	W159-6s	101.67	80.33	29.22	7.88	2.77	2.90	9.33	1793.87	194.63	0.88	1579.20	23.82	37.56
F_1	W161-6s	101.00	79.67	25.55	8.47	2.71	3.18	8.00	1264.83	154.59	0.55	577.33	24.02	14.54
F_1	W168-6s	102.67	95.33	29.77	8.29	2.66	3.18	9.43	1630.93	172.65	0.70	1144.43	23.58	27.09
F_1	W169-6s	97.67	79.67	27.78	8.21	2.58	3.26	9.37	1643.03	175.11	0.77	1272.73	23.76	30.28
F_1	W170-6s	104.67	80.00	27.53	8.39	2.64	3.25	10.33	1677.80	162.42	0.81	1369.97	24.18	33.15
F_1	W172-6s	105.67	79.00	28.12	8.58	2.53	3.44	8.10	1404.27	173.39	0.86	1207.40	24.86	30.05
F_1	W176-6s	98.67	88.67	27.38	7.87	2.76	2.89	9.20	1707.97	183.71	0.71	1281.83	22.90	29.08
F_1	W177-6s	101.00	85.00	27.77	7.64	2.69	2.90	10.00	1987.67	198.84	0.78	1553.47	22.00	34.19
F_1	W178-6s	120.00	96.33	26.26	7.71	2.58	3.05	11.20	2091.37	185.43	0.89	1875.83	21.37	40.41
F_1	W181-6s	111.33	88.67	29.53	8.24	2.59	3.23	11.25	1722.20	152.56	0.70	1211.10	23.36	28.36
F_1	W183-6s	121.33	88.33	28.29	8.72	2.49	3.60	6.50	1204.83	185.64	0.64	766.83	25.11	19.17
F_1	W184-6s	99.67	78.67	27.33	8.12	2.63	3.15	10.20	1466.63	143.48	0.66	952.97	23.97	22.82
F_1	W185-6s	102.00	79.67	27.75	8.49	2.50	3.51	9.33	1430.83	152.93	0.66	943.03	24.95	23.92
F_1	W190-6s	107.33	90.00	30.34	8.51	2.61	3.33	8.00	1462.50	182.81	0.77	1126.80	24.34	27.42
F_1	W191-6s	99.00	79.33	29.47	8.78	2.30	3.90	10.53	1691.43	159.94	0.72	1221.20	22.47	27.52
F_1	W194-6s	105.00	79.33	26.65	8.46	2.57	3.39	8.77	1549.33	176.54	0.69	1070.37	23.05	24.62
F_1	W199-6s	113.00	90.33	29.87	8.82	2.77	3.22	7.03	1218.10	172.83	0.78	949.00	26.89	25.47

F_1	W200-6s	107.67	88.00	27.86	8.28	2.57	3.30	9.20	1565.80	170.20	0.53	819.20	22.59	18.47
F_1	W221-6s	122.00	92.67	28.31	8.28	2.61	3.21	11.10	1945.47	174.98	0.82	1608.60	22.65	36.31
F_1	W222-6s	110.33	90.00	29.83	8.46	2.53	3.40	8.60	1514.00	176.05	0.71	1074.00	23.77	25.55
F_1	W223-6s	105.33	80.67	29.05	8.25	2.84	2.93	8.67	1608.60	185.18	0.65	1062.83	26.90	28.51
F_1	W224-6s	104.67	71.67	29.26	7.89	2.42	3.31	10.60	1914.27	180.57	0.83	1588.47	22.01	35.00
F_1	W225-6s	101.33	78.00	26.56	8.30	2.54	3.33	8.20	1407.53	171.70	0.64	904.77	22.89	20.73
F_1	W227-6s	105.67	79.67	27.27	8.61	2.58	3.45	11.63	1678.00	144.15	0.80	1347.03	25.14	34.03
F_1	W229-6s	118.67	87.33	29.71	8.48	2.50	3.48	8.13	1443.10	179.08	0.78	1123.83	23.19	26.09
F_1	W230-6s	114.00	94.33	29.48	8.30	2.69	3.13	9.50	1665.30	175.52	0.76	1271.83	22.64	29.23
F_1	W231-6s	120.67	79.67	28.19	8.14	2.67	3.09	10.25	1771.13	172.65	0.83	1471.00	23.25	33.81
F_1	W232-6s	104.00	80.67	26.79	7.62	2.62	2.95	11.10	2088.57	188.17	0.82	1711.50	21.42	36.65
F_1	W233-6s	105.33	79.33	27.46	7.55	2.73	2.80	9.75	2009.30	205.36	0.79	1593.10	22.94	36.50
F_1	W236-6s	100.67	80.00	29.84	8.47	2.64	3.27	8.07	1596.30	198.41	0.80	1282.10	24.86	31.83
F_1	W240-6s	105.00	78.33	26.87	8.60	2.74	3.19	11.03	1698.07	154.01	0.75	1289.43	26.32	33.90
F_1	W242-6s	106.67	91.67	27.87	8.35	2.65	3.21	13.07	2229.33	170.85	0.68	1511.13	24.25	36.68
F_1	W263-6s	107.67	94.00	28.54	7.71	2.71	2.90	8.25	1723.30	208.95	0.85	1478.97	20.08	29.86
F_1	W265-6s	132.33	92.67	29.96	7.72	2.71	2.90	14.30	2759.00	192.94	0.88	2410.00	21.17	50.87
F_1	W267-6s	103.00	71.33	26.10	8.42	2.66	3.22	9.10	1448.30	158.41	0.88	1270.47	24.96	31.74
F_1	W269-6s	104.00	79.00	28.23	8.33	2.55	3.33	8.70	1514.10	172.85	0.80	1204.60	22.31	26.89
F_1	W270-6s	97.00	75.67	23.77	7.37	2.70	2.80	12.53	1796.33	144.55	0.69	1194.70	22.07	26.34
F_1	W293-6s	112.00	87.33	27.63	8.64	2.73	3.24	9.03	1619.83	179.03	0.77	1247.67	26.29	32.81
F_1	W295-6s	110.67	90.67	30.22	8.43	2.51	3.40	10.40	1647.40	158.69	0.58	941.57	23.91	22.53
F_1	W298-6s	96.00	74.00	27.28	8.24	2.56	3.27	9.43	1545.93	164.70	0.75	1164.27	23.37	27.21
F_1	W301-6s	104.33	86.00	28.81	8.65	2.55	3.47	12.70	1888.40	149.10	0.61	1164.23	24.23	28.07
F_1	W304-6s	104.00	86.00	28.80	7.93	2.68	3.00	9.50	2312.50	243.42	0.57	1310.50	22.32	29.26
F_1	W308-6s	112.67	92.67	30.48	8.40	2.55	3.40	10.83	1979.50	179.94	0.77	1518.17	24.33	37.23
F_1	W309-6s	108.33	84.67	30.22	8.44	2.50	3.43	10.67	1849.17	173.53	0.82	1509.50	23.08	35.10
F_1	W310-6s	112.00	88.00	29.97	8.17	2.52	3.30	10.87	1907.70	174.64	0.78	1498.10	21.60	32.51
F_1	W311-6s	102.33	93.33	28.04	8.21	2.54	3.27	9.47	1650.30	172.44	0.83	1367.67	23.14	31.79
F_1	W312-6s	110.00	90.33	28.14	8.45	2.54	3.40	10.50	1597.00	152.10	0.58	884.00	23.64	20.85

F_1	W315-6s	101.67	89.33	30.16	8.23	2.51	3.33	10.23	1842.00	178.47	0.68	1257.40	23.03	29.11
F_1	W317-6s	112.33	90.33	27.74	8.63	2.63	3.42	9.30	1442.00	155.05	0.75	1070.80	23.20	24.85
F_1	R287-6s	111.00	73.00	27.94	8.25	2.51	3.37	8.30	1623.70	195.63	0.81	1332.30	23.02	30.76
F_1	6-815s	107.00	112.00	26.02	7.83	2.38	3.33	10.97	1936.80	176.92	0.71	1367.73	20.80	28.41
F_1	7-815s	105.33	80.33	25.51	7.84	2.40	3.33	10.93	2098.67	191.45	0.73	1556.33	20.62	32.80
F_1	10-815s	107.00	79.33	26.24	8.71	2.55	3.47	8.00	1568.70	196.09	0.59	934.70	24.31	22.68
F_1	31-815s	105.33	89.67	26.49	8.75	2.58	3.47	7.50	1530.70	205.37	0.70	1076.20	24.99	27.05
F_1	36-815s	115.67	89.00	23.33	9.05	2.72	3.40	9.40	1553.00	165.21	0.68	1048.20	28.54	29.87
F_1	37-815s	115.33	83.33	24.77	8.02	2.60	3.16	7.93	1490.90	186.42	0.65	980.00	22.94	22.70
F_1	38-815s	129.00	88.00	29.22	8.60	2.61	3.36	11.27	2354.50	215.02	0.63	1491.83	24.93	37.34
F_1	41-815s	109.00	79.00	26.47	7.75	2.83	2.77	10.17	2240.60	219.57	0.79	1792.00	23.39	42.20
F_1	43-815s	115.33	88.67	25.90	8.07	2.74	3.00	7.83	1463.60	188.92	0.76	1103.00	25.23	27.68
F_1	44-815s	119.00	93.00	26.25	8.99	2.56	3.57	7.50	1544.17	207.61	0.64	976.67	25.67	25.31
F_1	45-815s	104.00	80.33	24.45	7.32	2.35	3.18	10.50	2610.73	248.34	0.76	1954.73	17.55	34.13
F_1	46-815s	105.33	79.67	27.46	8.58	2.54	3.40	12.00	2367.50	198.57	0.71	1678.77	23.58	39.42
F_1	49-815s	115.67	89.67	28.31	8.95	2.57	3.58	9.80	1746.43	176.41	0.60	1027.60	27.40	28.27
F_1	50-815s	101.00	85.00	25.16	8.93	2.52	3.60	10.00	1776.00	177.60	0.74	1320.00	23.94	31.60
F_1	53-815s	129.00	76.00	28.00	8.67	2.56	3.46	11.10	2124.93	191.16	0.64	1345.77	26.30	35.13
F_1	60-815s	105.00	87.00	26.21	8.12	2.59	3.23	9.50	1741.67	182.83	0.67	1202.50	23.38	28.08
F_1	61-815s	105.00	144.33	NA	NA	NA	NA	NA	NA	NA	NA	NA	NA	NA
F_1	118-815s	107.33	76.00	27.49	8.49	2.44	3.55	11.00	2348.00	213.45	0.64	1473.50	23.75	34.98
F_1	147-815s	107.00	89.67	25.92	8.49	2.53	3.44	9.73	1892.15	194.44	0.66	1246.66	24.67	30.61
F_1	148-815s	119.00	96.67	23.60	9.11	2.45	3.80	7.00	1313.00	187.57	0.49	649.00	26.36	17.11
F_1	151-815s	131.00	88.00	27.14	8.08	2.67	3.06	10.40	2576.57	243.60	0.79	2071.27	23.46	48.62
F_1	165-815s	111.67	85.67	27.16	7.90	2.81	2.85	12.75	2714.00	213.08	0.74	2025.27	24.00	48.64
F_1	166-815s	105.33	80.00	26.08	7.89	2.60	3.09	9.27	1787.70	190.53	0.77	1363.27	21.80	29.99
F_1	169-815s	131.33	89.33	28.46	8.02	2.74	3.00	11.17	2329.00	205.86	0.81	1906.57	24.84	47.51
F_1	170-815s	99.33	79.00	26.73	7.65	2.81	2.77	12.17	1782.17	146.41	0.86	1527.33	23.99	36.60
F_1	173-815s	120.33	85.33	26.78	7.71	2.62	3.00	9.10	2032.40	225.66	0.67	1376.50	22.76	31.35
F_1	175-815s	113.33	85.00	25.55	8.92	2.44	3.75	10.50	1885.47	179.68	0.78	1460.60	24.95	36.35

F_1	178-815s	112.33	81.67	27.33	7.96	2.63	3.10	14.17	2976.67	209.30	0.77	2295.33	23.66	54.28
F_1	W017-815s	109.00	84.33	22.73	8.85	2.40	3.78	11.80	1956.50	165.81	0.61	1187.00	21.80	25.94
F_1	W040-815s	119.00	85.00	27.11	8.75	2.48	3.61	10.77	2178.93	202.68	0.42	940.00	24.89	23.58
F_1	W044-815s	109.67	88.00	26.85	8.74	2.58	3.43	8.40	1548.27	185.04	0.61	938.27	26.48	24.82
F_1	W046-815s	121.00	95.00	24.76	8.31	2.68	3.16	8.77	1518.17	174.78	0.71	1078.90	23.82	25.81
F_1	W049-815s	152.00	129.67	26.50	8.75	2.53	3.50	17.50	2547.00	145.54	0.44	1109.00	24.36	27.01
F_1	W066-815s	134.67	144.00	NA	NA	NA	NA	NA	NA	NA	NA	NA	NA	NA
F_1	W078-815s	110.00	144.67	NA	NA	NA	NA	NA	NA	NA	NA	NA	NA	NA
F_1	W083-815s	116.00	141.33	NA	NA	NA	NA	NA	NA	NA	NA	NA	NA	NA
F_1	W086-815s	110.00	80.00	25.96	8.02	2.40	3.43	8.60	1952.00	226.98	0.74	1427.80	21.33	30.44
F_1	W118-815s	117.33	76.00	26.88	9.20	2.43	3.85	7.00	1506.00	215.14	0.84	1262.00	27.63	34.89
F_1	W137-815s	110.00	142.33	NA	NA	NA	NA	NA	NA	NA	NA	NA	NA	NA
F_1	W138-815s	102.33	79.67	24.70	7.83	2.49	3.20	11.07	2077.53	186.50	0.74	1567.83	22.05	34.43
F_1	W147-815s	110.00	80.00	26.95	8.48	2.59	3.36	11.75	2203.67	187.86	0.55	1218.67	24.80	30.11
F_1	W149-815s	103.33	77.00	27.23	8.93	2.75	3.33	10.83	1598.33	148.08	0.46	733.00	25.15	18.44
F_1	W153-815s	99.67	80.00	25.49	9.03	2.59	3.55	9.40	1687.03	178.94	0.79	1329.97	25.79	34.11
F_1	W156-815s	113.33	80.33	26.30	7.61	2.62	2.94	9.00	1989.20	221.02	0.61	1221.00	22.47	26.97
F_1	W159-815s	111.33	80.00	24.60	8.10	2.74	3.00	11.27	2075.57	186.04	0.87	1832.30	24.10	44.21
F_1	W161-815s	104.33	79.33	25.70	8.12	2.55	3.22	10.13	2187.47	214.25	0.80	1772.63	22.96	40.31
F_1	W168-815s	112.67	96.33	28.64	8.77	2.73	3.30	11.93	2188.17	183.67	0.71	1542.90	30.01	47.00
F_1	W169-815s	97.67	75.33	26.40	8.59	2.50	3.49	10.90	1911.30	172.90	0.71	1330.97	25.17	33.51
F_1	W170-815s	111.00	80.00	26.40	8.05	2.59	3.14	9.77	2017.27	207.74	0.79	1598.10	22.85	36.62
F_1	W172-815s	111.33	80.33	27.27	8.70	2.41	3.70	10.05	1912.37	190.37	0.72	1387.70	24.07	33.21
F_1	W176-815s	107.00	137.67	NA	NA	NA	NA	NA	NA	NA	NA	NA	NA	NA
F_1	W177-815s	101.00	133.67	25.72	7.85	2.68	3.00	9.33	1610.17	173.22	0.73	1172.50	22.96	26.93
F_1	W178-815s	122.00	143.00	NA	NA	NA	NA	NA	NA	NA	NA	NA	NA	NA
F_1	W181-815s	109.67	86.67	27.37	8.50	2.49	3.50	11.40	1867.20	162.89	0.63	1175.93	24.80	29.12
F_1	W183-815s	111.33	88.00	23.05	7.86	2.47	3.25	6.50	1073.67	165.82	0.71	737.00	20.88	15.55
F_1	W184-815s	104.67	76.33	25.98	8.40	2.53	3.39	11.83	1669.13	142.31	0.63	1059.60	24.54	25.88
F_1	W185-815s	105.67	78.67	26.84	8.73	2.44	3.69	11.23	1703.10	150.72	0.59	987.73	25.24	24.88

F_1	W190-815s	115.00	91.67	27.91	8.72	2.58	3.45	8.83	1347.43	159.85	0.66	888.77	25.85	22.94
F_1	W191-815s	106.00	79.33	27.21	8.96	2.25	4.11	11.57	1839.03	160.04	0.70	1290.93	22.97	29.64
F_1	W194-815s	114.33	79.67	24.76	8.70	2.51	3.53	7.17	1351.50	185.01	0.57	774.67	24.02	18.85
F_1	W199-815s	115.67	80.33	27.45	8.57	2.72	3.20	7.65	1575.17	205.81	0.56	889.17	26.59	23.67
F_1	W200-815s	103.00	133.33	26.70	9.25	2.48	3.80	7.00	1335.50	190.79	0.85	1133.50	27.35	31.01
F_1	W221-815s	119.00	145.67	28.68	7.35	1.98	3.80	9.50	1849.00	194.63	0.85	1571.50	14.99	23.55
F_1	W222-815s	103.00	90.33	24.76	8.46	2.50	3.45	8.63	1298.03	164.98	0.43	550.87	20.78	11.70
F_1	W223-815s	110.33	80.67	26.80	8.31	2.74	3.10	10.83	2199.67	201.13	0.59	1344.17	25.99	34.86
F_1	W224-815s	127.33	82.67	28.10	7.53	2.46	3.10	9.70	2215.30	228.38	0.65	1435.30	20.72	29.70
F_1	W225-815s	112.67	77.67	27.56	8.80	2.55	3.53	10.50	2411.27	227.79	0.52	1294.40	24.72	31.74
F_1	W227-815s	107.00	76.00	26.07	8.88	2.47	3.67	10.57	1779.00	168.66	0.77	1370.13	26.23	35.82
F_1	W229-815s	118.00	85.33	28.70	8.76	2.44	3.66	8.17	1581.83	193.73	0.65	1026.23	24.99	25.56
F_1	W230-815s	109.00	129.67	27.02	9.01	2.39	3.90	8.00	1790.00	223.75	0.71	1267.00	25.77	32.66
F_1	W231-815s	121.67	86.00	28.00	8.08	2.66	3.09	9.77	1698.20	173.92	0.83	1417.80	24.00	34.03
F_1	W232-815s	108.00	86.67	26.24	7.67	2.51	3.12	10.13	2134.13	210.65	0.72	1555.81	21.44	33.36
F_1	W233-815s	117.67	88.67	26.71	8.65	2.73	3.30	11.00	2549.00	231.73	0.80	2047.50	26.10	52.98
F_1	W236-815s	103.67	80.00	27.49	8.75	2.61	3.40	8.30	1945.10	234.89	0.80	1544.33	24.58	37.37
F_1	W237-815s	110.67	82.67	27.46	8.57	2.48	3.55	10.33	2244.53	217.95	0.70	1571.70	23.34	36.89
F_1	W240-815s	114.00	79.00	22.77	8.90	2.73	3.30	9.00	1367.00	151.03	0.32	444.83	28.07	12.46
F_1	W242-815s	113.33	88.33	25.04	8.44	2.65	3.25	13.85	2125.00	153.63	0.35	757.50	23.99	17.67
F_1	W263-815s	121.67	83.67	25.99	8.26	2.56	3.31	7.83	1507.87	191.41	0.71	1104.70	22.17	24.74
F_1	W265-815s	129.00	145.00	NA	NA	NA	NA	NA	NA	NA	NA	NA	NA	NA
F_1	W267-815s	112.67	73.33	25.98	8.63	2.61	3.38	8.33	1465.07	172.47	0.55	924.37	24.18	22.75
F_1	W270-815s	130.33	80.33	23.14	7.53	2.63	2.90	11.83	3061.90	256.32	0.80	2437.17	21.46	51.97
F_1	W293-815s	101.00	87.67	25.72	8.53	2.71	3.18	5.00	889.83	178.25	0.70	611.10	25.10	15.32
F_1	W295-815s	114.67	90.67	26.89	8.70	2.46	3.60	9.97	1682.37	171.20	0.70	1230.00	25.27	30.76
F_1	W298-815s	102.00	75.33	23.81	8.09	2.49	3.27	13.50	2293.23	175.52	0.68	1504.70	22.39	33.81
F_1	W301-815s	101.67	92.67	27.12	9.03	2.41	3.83	6.33	983.67	154.86	0.48	473.83	25.28	11.95
F_1	W304-815s	109.00	78.00	26.36	8.04	2.63	3.13	11.30	2771.80	245.29	0.56	1646.50	20.35	32.96
F_1	W308-815s	119.67	144.00	NA	NA	NA	NA	NA	NA	NA	NA	NA	NA	NA

F_1	W309-815s	118.33	143.00	NA	NA	NA	NA	NA	NA	NA	NA	NA	NA	NA
F_1	W310-815s	125.00	88.00	27.77	8.48	2.39	3.59	8.35	1566.57	187.53	0.69	1111.40	23.80	26.48
F_1	W311-815s	114.33	87.33	26.35	8.38	2.42	3.52	9.40	1976.40	209.56	0.72	1422.47	22.64	32.23
F_1	W312-815s	112.00	88.00	26.97	8.43	2.49	3.47	8.70	1545.63	177.28	0.58	911.83	24.50	22.18
F_1	W315-815s	112.33	88.00	24.69	8.77	2.50	3.60	9.50	1881.33	196.95	0.80	1480.33	24.84	36.58
F_1	W317-815s	126.00	92.33	26.19	8.64	2.52	3.50	8.10	1731.33	214.35	0.74	1309.27	24.75	33.35
F_1	R287-815s	120.67	76.33	26.84	8.19	2.53	3.32	8.60	1990.67	237.37	0.77	1544.77	22.18	34.63
F_1	6-9802s	106.67	78.00	22.48	7.91	2.38	3.38	11.60	2156.63	185.87	0.57	1191.77	20.12	23.95
F_1	7-9802s	104.33	79.00	23.36	7.81	2.35	3.38	13.27	1964.07	152.39	0.60	1184.93	19.86	23.58
F_1	10-9802s	106.67	105.67	25.29	8.68	2.49	3.58	5.80	1447.40	249.55	0.75	1092.40	25.08	27.39
F_1	31-9802s	104.33	83.67	23.82	9.11	2.49	3.80	10.00	1763.00	176.30	0.51	906.00	24.80	22.47
F_1	36-9802s	91.00	71.00	20.40	8.88	2.51	3.57	10.70	1326.00	123.93	0.68	876.30	25.59	22.45
F_1	37-9802s	101.67	72.67	22.75	7.78	2.60	3.07	12.77	1599.90	130.02	0.60	963.13	21.89	21.19
F_1	38-9802s	104.00	53.67	23.82	8.33	2.41	3.55	10.60	1516.87	143.92	0.74	1123.90	22.24	24.96
F_1	41-9802s	105.93	85.00	19.10	7.78	2.62	3.00	23.00	2326.00	101.13	0.73	1686.50	21.93	36.98
F_1	43-9802s	108.33	79.33	22.60	8.12	2.73	3.00	13.00	2220.17	171.65	0.79	1754.70	25.25	44.29
F_1	44-9802s	107.67	88.33	25.10	9.10	2.56	3.65	5.00	1111.67	232.55	0.81	903.03	26.71	24.35
F_1	45-9802s	111.67	82.33	21.93	7.77	2.37	3.37	8.17	1689.43	208.05	0.87	1471.17	19.82	29.22
F_1	46-9802s	95.00	76.00	23.68	8.71	2.42	3.70	10.50	1692.80	161.22	0.75	1277.80	22.86	29.17
F_1	49-9802s	104.67	79.00	24.42	9.04	2.44	3.80	8.20	1515.03	189.52	0.69	1028.83	25.54	26.11
F_1	50-9802s	108.00	80.33	23.99	9.10	2.41	3.88	11.85	2142.30	180.49	0.83	1789.30	23.22	41.39
F_1	53-9802s	120.00	76.67	24.47	9.24	2.62	3.60	11.67	1922.50	166.49	0.69	1341.17	28.97	38.95
F_1	60-9802s	110.33	84.33	24.12	8.08	2.48	3.33	12.77	2533.10	196.65	0.69	1776.33	21.70	38.79
F_1	61-9802s	110.00	142.33	NA	NA	NA	NA	NA	NA	NA	NA	NA	NA	NA
F_1	118-9802s	110.00	144.00	NA	NA	NA	NA	NA	NA	NA	NA	NA	NA	NA
F_1	147-9802s	109.00	78.00	23.25	9.08	2.52	3.69	11.77	1859.93	158.13	0.82	1528.23	25.57	39.17
F_1	148-9802s	120.67	98.00	25.37	8.98	2.45	3.76	9.00	2210.87	241.93	0.79	1774.20	25.82	46.35
F_1	164-9802s	101.00	68.33	24.09	8.19	2.65	3.14	12.67	1684.17	133.41	0.65	1103.83	23.77	26.19
F_1	165-9802s	100.33	70.67	22.61	7.99	2.62	3.10	13.57	1844.63	141.11	0.60	1102.47	23.00	25.41
F_1	166-9802s	105.00	78.00	24.37	8.57	2.56	3.45	9.07	1597.67	176.13	0.81	1294.33	24.46	31.58

F_1	169-9802s	110.33	81.00	24.80	7.89	2.57	3.10	20.00	3279.00	163.95	0.78	2569.00	23.23	59.68
F_1	170-9802s	99.00	76.67	23.75	7.96	2.66	3.05	12.17	1524.43	124.47	0.76	1157.93	23.26	26.91
F_1	173-9802s	115.00	78.33	23.63	7.94	2.64	3.07	10.92	1881.67	171.18	0.66	1268.33	23.56	29.74
F_1	175-9802s	116.00	83.33	22.88	8.80	2.34	3.85	11.30	2146.40	189.95	0.77	1660.30	22.61	37.74
F_1	178-9802s	105.33	80.00	23.25	8.05	2.64	3.10	9.47	1610.33	171.36	0.79	1274.90	23.87	30.42
F_1	W040-9802s	110.00	141.00	NA	NA	NA	NA	NA	NA	NA	NA	NA	NA	NA
F_1	W044-9802s	101.00	86.67	23.66	8.98	2.47	3.72	10.10	1709.83	170.60	0.74	1280.27	26.64	34.12
F_1	W046-9802s	104.67	83.33	24.41	8.53	2.60	3.33	10.07	1922.63	191.41	0.74	1432.03	24.33	34.92
F_1	W049-9802s	157.00	119.33	26.48	8.03	2.34	3.49	10.96	2139.10	195.14	0.83	1769.60	20.08	36.02
F_1	W066-9802s	132.00	131.67	NA	NA	NA	NA	NA	NA	NA	NA	NA	NA	NA
F_1	W078-9802s	105.00	141.67	NA	NA	NA	NA	NA	NA	NA	NA	NA	NA	NA
F_1	W083-9802s	113.00	133.67	NA	NA	NA	NA	NA	NA	NA	NA	NA	NA	NA
F_1	W086-9802s	111.00	79.33	22.41	8.81	2.47	3.65	13.75	2340.00	169.98	0.74	1738.00	23.52	40.73
F_1	W118-9802s	126.33	105.00	23.42	9.30	2.52	3.76	8.00	1505.20	188.15	0.16	242.00	23.99	5.83
F_1	W137-9802s	122.00	106.67	23.56	9.04	2.78	3.30	8.20	1446.60	176.41	0.62	890.60	27.18	24.16
F_1	W138-9802s	100.00	65.67	19.81	7.77	2.34	3.40	13.90	1652.93	119.85	0.77	1281.63	19.80	25.46
F_1	W147-9802s	100.00	78.00	23.31	8.86	2.54	3.53	8.30	1303.70	157.07	0.62	802.30	24.67	19.77
F_1	W149-9802s	112.33	77.00	23.03	9.16	2.62	3.61	11.37	1689.30	149.63	0.58	983.17	25.07	24.69
F_1	W153-9802s	100.67	81.67	24.54	9.00	2.54	3.63	9.43	1365.27	144.97	0.84	1149.00	25.47	29.26
F_1	W156-9802s	122.67	78.33	26.70	7.84	2.76	2.87	9.50	1957.70	206.07	0.72	1402.70	25.59	35.62
F_1	W159-9802s	110.33	76.00	23.27	8.32	2.58	3.30	12.07	1920.63	160.79	0.91	1740.93	23.63	41.10
F_1	W161-9802s	104.67	65.67	20.12	8.61	2.47	3.60	8.00	1367.00	170.88	0.84	1151.00	24.43	28.12
F_1	W168-9802s	102.00	96.67	27.10	8.44	2.60	3.30	9.00	1599.50	177.72	0.40	645.50	27.20	17.55
F_1	W169-9802s	105.00	74.67	22.59	8.55	2.47	3.53	9.80	1584.00	161.72	0.72	1156.27	24.15	28.20
F_1	W170-9802s	111.00	82.33	23.37	8.83	2.49	3.65	10.03	1792.50	178.77	0.73	1341.33	24.53	32.87
F_1	W172-9802s	111.00	77.33	25.19	8.92	2.37	3.87	10.23	1828.83	179.34	0.75	1379.20	25.17	34.72
F_1	W176-9802s	112.00	129.67	27.30	8.83	2.65	3.40	13.00	2523.50	194.12	0.70	1755.50	26.91	47.25
F_1	W177-9802s	109.00	129.67	23.75	7.96	2.62	3.13	10.66	1946.53	182.57	0.75	1453.57	22.60	33.25
F_1	W178-9802s	119.65	138.67	NA	NA	NA	NA	NA	NA	NA	NA	NA	NA	NA
F_1	W181-9802s	111.00	141.33	NA	NA	NA	NA	NA	NA	NA	NA	NA	NA	NA

F_1	W183-9802s	113.00	143.00	NA	NA	NA	NA	NA	NA	NA	NA	NA	NA	NA
F_1	W184-9802s	101.00	79.67	25.20	8.70	2.48	3.58	12.17	1665.93	166.09	0.60	996.57	24.50	24.64
F_1	W185-9802s	98.67	76.33	23.61	8.77	2.34	3.84	10.17	1386.60	138.36	0.68	948.53	24.05	22.84
F_1	W194-9802s	111.00	71.67	19.93	8.83	2.34	3.87	14.70	1522.30	103.56	0.59	908.00	21.68	19.67
F_1	W199-9802s	103.33	90.00	24.33	8.97	2.47	3.73	8.00	1555.50	194.44	0.79	1221.00	25.59	31.34
F_1	W200-9802s	119.33	106.33	26.16	8.91	2.42	3.80	9.25	1775.50	192.41	0.73	1300.27	23.10	30.06
F_1	W221-9802s	115.33	95.67	24.25	8.37	2.58	3.30	8.33	1416.37	169.99	0.84	1181.83	22.66	26.77
F_1	W222-9802s	116.00	133.67	NA	NA	NA	NA	NA	NA	NA	NA	NA	NA	NA
F_1	W223-9802s	116.00	79.33	24.03	8.65	2.68	3.30	9.43	1838.27	195.57	0.54	993.73	26.40	26.33
F_1	W224-9802s	120.33	70.00	24.13	8.40	2.32	3.72	14.33	1852.37	131.20	0.72	1365.83	22.46	30.92
F_1	W227-9802s	113.33	76.33	24.59	8.99	2.37	3.89	15.27	2024.30	135.70	0.63	1267.73	25.05	31.81
F_1	W229-9802s	120.00	67.67	27.03	8.91	2.33	3.93	10.83	2128.13	194.02	0.60	1281.13	23.77	30.55
F_1	W230-9802s	110.00	134.67	NA	NA	NA	NA	NA	NA	NA	NA	NA	NA	NA
F_1	W231-9802s	114.33	96.00	24.88	8.24	2.60	3.22	8.80	1601.40	181.98	0.74	1202.20	22.39	27.18
F_1	W233-9802s	112.00	77.67	21.99	7.91	2.62	3.07	8.50	2126.30	250.15	0.78	1601.70	22.94	36.76
F_1	W236-9802s	112.00	76.67	25.65	8.95	2.52	3.64	10.33	2128.03	205.33	0.81	1730.70	25.33	43.48
F_1	W237-9802s	110.00	143.00	NA	NA	NA	NA	NA	NA	NA	NA	NA	NA	NA
F_1	W240-9802s	123.00	105.00	23.96	8.60	2.34	3.80	6.00	1455.00	242.50	0.83	1206.00	24.79	29.90
F_1	W242-9802s	94.67	81.00	22.44	8.71	2.49	3.58	13.90	1635.97	117.70	0.38	596.97	24.43	14.61
F_1	W263-9802s	115.00	141.00	NA	NA	NA	NA	NA	NA	NA	NA	NA	NA	NA
F_1	W265-9802s	123.00	132.67	NA	NA	NA	NA	NA	NA	NA	NA	NA	NA	NA
F_1	W267-9802s	115.33	75.00	21.93	8.68	2.52	3.56	9.90	2235.67	222.99	0.70	1502.57	24.76	37.20
F_1	W269-9802s	92.00	65.33	20.12	8.63	2.28	3.90	20.25	1975.50	98.56	0.74	1479.50	20.77	30.77
F_1	W270-9802s	109.00	75.00	20.46	7.66	2.53	3.09	10.10	1758.97	174.10	0.89	1563.00	21.97	34.32
F_1	W293-9802s	107.00	87.00	24.18	8.61	2.58	3.43	9.17	1590.27	173.63	0.62	996.20	25.21	25.10
F_1	W295-9802s	103.00	142.33	NA	NA	NA	NA	NA	NA	NA	NA	NA	NA	NA
F_1	W298-9802s	101.00	76.00	23.16	8.67	2.47	3.60	13.00	1934.77	148.10	0.74	1447.43	23.44	34.05
F_1	W301-9802s	100.00	139.00	NA	NA	NA	NA	NA	NA	NA	NA	NA	NA	NA
F_1	W308-9802s	120.00	131.33	NA	NA	NA	NA	NA	NA	NA	NA	NA	NA	NA
F_1	W309-9802s	116.00	141.00	NA	NA	NA	NA	NA	NA	NA	NA	NA	NA	NA

F_1	W310-9802s	118.00	140.67	NA	NA	NA	NA	NA	NA	NA	NA	NA	NA	NA
F_1	W311-9802s	113.00	85.33	23.78	8.64	2.41	3.69	10.90	2159.87	198.40	0.72	1553.60	23.75	36.88
F_1	W312-9802s	103.00	133.00	NA	NA	NA	NA	NA	NA	NA	NA	NA	NA	NA
F_1	W315-9802s	94.00	134.33	NA	NA	NA	NA	NA	NA	NA	NA	NA	NA	NA
F_1	W317-9802s	118.33	139.33	21.80	8.54	2.54	3.40	9.00	1487.00	165.22	0.72	1069.00	23.57	25.20
F_1	R287-9802s	110.67	66.33	23.67	8.78	2.32	3.87	19.00	2556.67	134.75	0.68	1728.33	22.54	38.94
